# Mice deficient in synaptic protease neurotrypsin show impaired spaced long-term potentiation and blunted learning-induced modulation of dendritic spines

**DOI:** 10.1007/s00018-023-04720-z

**Published:** 2023-03-05

**Authors:** Maura Ferrer-Ferrer, Shaobo Jia, Rahul Kaushik, Jenny Schneeberg, Izabela Figiel, Stepan Aleshin, Andrey Mironov, Motahareh Safari, Renato Frischknecht, Jakub Wlodarczyk, Oleg Senkov, Alexander Dityatev

**Affiliations:** 1 Molecular Neuroplasticity Group, German Center for Neurodegenerative Diseases (DZNE), 39120 Magdeburg, Germany; 2 Nencki Institute of Experimental Biology, Polish Academy of Sciences, Pasteura 3, 02-093 Warsaw, Poland; 3 Department of Biology, Animal Physiology, Friedrich-Alexander-Universität Erlangen-Nürnberg, 91058 Erlangen, Germany; 4 Center for Behavioral Brain Sciences (CBBS), 39106 Magdeburg, Germany; 5 Medical Faculty, Otto-Von-Guericke University, 39120 Magdeburg, Germany

**Keywords:** Extracellular proteolysis, Extracellular matrix, Filopodia, Dendritic spine, Synaptic plasticity, Learning and memory, Hebbian

## Abstract

Neurotrypsin (NT) is a neuronal trypsin-like serine protease whose mutations cause severe mental retardation in humans. NT is activated in vitro by Hebbian-like conjunction of pre- and postsynaptic activities, which promotes the formation of dendritic filopodia via proteolytic cleavage of the proteoglycan agrin. Here, we investigated the functional importance of this mechanism for synaptic plasticity, learning, and extinction of memory. We report that juvenile neurotrypsin-deficient (NT^−/−^) mice exhibit impaired long-term potentiation induced by a spaced stimulation protocol designed to probe the generation of new filopodia and their conversion into functional synapses. Behaviorally, juvenile NT^−/−^ mice show impaired contextual fear memory and have a sociability deficit. The latter persists in aged NT^−/−^ mice, which, unlike juvenile mice, show normal recall but impaired extinction of contextual fear memories. Structurally, juvenile mutants exhibit reduced spine density in the CA1 region, fewer thin spines, and no modulation in the density of dendritic spines following fear conditioning and extinction in contrast to wild-type littermates. The head width of thin spines is reduced in both juvenile and aged NT^−/−^ mice. In vivo delivery of adeno-associated virus expressing an NT-generated fragment of agrin, agrin-22, but not a shorter agrin-15, elevates the spine density in NT^−/−^ mice. Moreover, agrin-22 co-aggregates with pre- and postsynaptic markers and increases the density and size of presynaptic boutons and presynaptic puncta, corroborating the view that agrin-22 supports the synaptic growth.

**Supplementary Information:**

The online version contains supplementary material available at 10.1007/s00018-023-04720-z.

## Introduction

Multiple studies have implicated the role of extracellular proteases in synaptic plasticity, learning, and memory [1, 2]. Neurotrypsin (NT), a neuronal trypsin-like serine protease, has been recognized to play an essential role in cognitive brain function due to a 4-nucleotide deletion in the PRSS12 gene of the human chromosome 4q, which results in an earlier stop codon and the production of a truncated protein leading to severe mental retardation in humans [3]. In the adult central nervous system (CNS) of humans, NT mRNA is highly expressed in the hippocampus (particularly in the subiculum and pyramidal cells of the CA1 region), cerebral cortex, and amygdala [4]. In the developing mouse brain, postnatal NT mRNA is strongly expressed in the cortex and hippocampus, reaching its peak expression during neural development and correlating with periods of synaptogenesis [5]. By electron microscopy, NT was localized at the presynaptic terminals of human cortical synapses [3]. Live imaging studies in cultured hippocampal neurons revealed that NT is recruited and released presynaptically in an activity-dependent manner [6]. Interestingly, the proteolytic activity of NT requires coincident activation of postsynaptic NMDA receptors, i.e., its activation presumably occurs in a Hebbian manner [7]. At present, the only known proteolytic substrate of NT is the proteoglycan agrin. Synaptic agrin is cleaved by NT at two sites, yielding a 110 kDa N-terminal fragment, a 90 kDa internal fragment, and a 22 kDa C-terminal fragment [8]. In rodent brain slices, activity-dependent exocytosis of NT from presynaptic terminals—followed by agrin cleavage—induces the formation of dendritic filopodia. This is in line with the finding that in NT knockout (NT^−/−^) brain slices, no activity-dependent generation of dendritic filopodia is observed. However, activity-dependent formation of filopodia in slices could be rescued by application of agrin-22 but not agrin-90 fragments. A shorter version of agrin-22, agrin-15, was shown to lack agrin-mediated activity in hippocampal and cortical cultures [9] and acute-slice preparations [10]. The clinical relevance of this signaling mechanism is supported by a recent study, showing that in a mouse model of infantile neuronal ceroid lipofuscinosis, in which the mice are deficient in ceroid lipofuscinosis neuronal-1 (Cln1), NT activity is suppressed due to upregulation of its inhibitor serpina1. A deficit in agrin-22 in this mutant may explain the synaptic dysfunctions characteristic of this disease [11].

Previous work has reported that the level of conventional theta-burst stimulation (TBS)-induced LTP is normal in hippocampal slices from NT^−/−^ mice [7]. However, the conventional protocol is not suited to detect the enhancement of synaptic transmission due to the formation of new spines, as nascent spines are mostly silent due to a lack of AMPA receptors [12–15]. It is plausible to assume, however, that the newly generated silent synapses induced by agrin in response to the first TBS of presynaptic axons could become potentiated in the response to the second TBS applied 1 h after the first TBS. This time interval is necessary for the recruitment of postsynaptic density components to nascent synapses, which can be used as a scaffold for the recruitment of AMPA receptors [16]. Importantly, maturation of activity-induced filopodia-like dendritic protrusions into stable and functional spines with postsynaptic density can be observed in vivo and is essential for learning and memory formation [17].

Here, we investigated the functional importance of NT in this process in vivo. We found that NT^−/−^ mice have an impairment of a specific form of long-term potentiation dependent on filopodia generation and their conversion into functional synapses. Behaviorally, NT^−/−^ mice showed deficits in contextual fear memory and sociability compared to their NT^+/+^ littermates, suggesting an essential role of NT in regulating multiple aspects of higher cognitive functions in the brain. Morphological analysis revealed significantly reduced spine density in NT^−/−^ mice. Furthermore, NT^−/−^ mice have shown a lower proportion of thin/filopodia-like spines, in line with the hypothesis that NT may specifically affect filopodia formation.

## Results

### Contribution of neurotrypsin (NT) to synaptic plasticity in juvenile mice

**Fig. 1:**
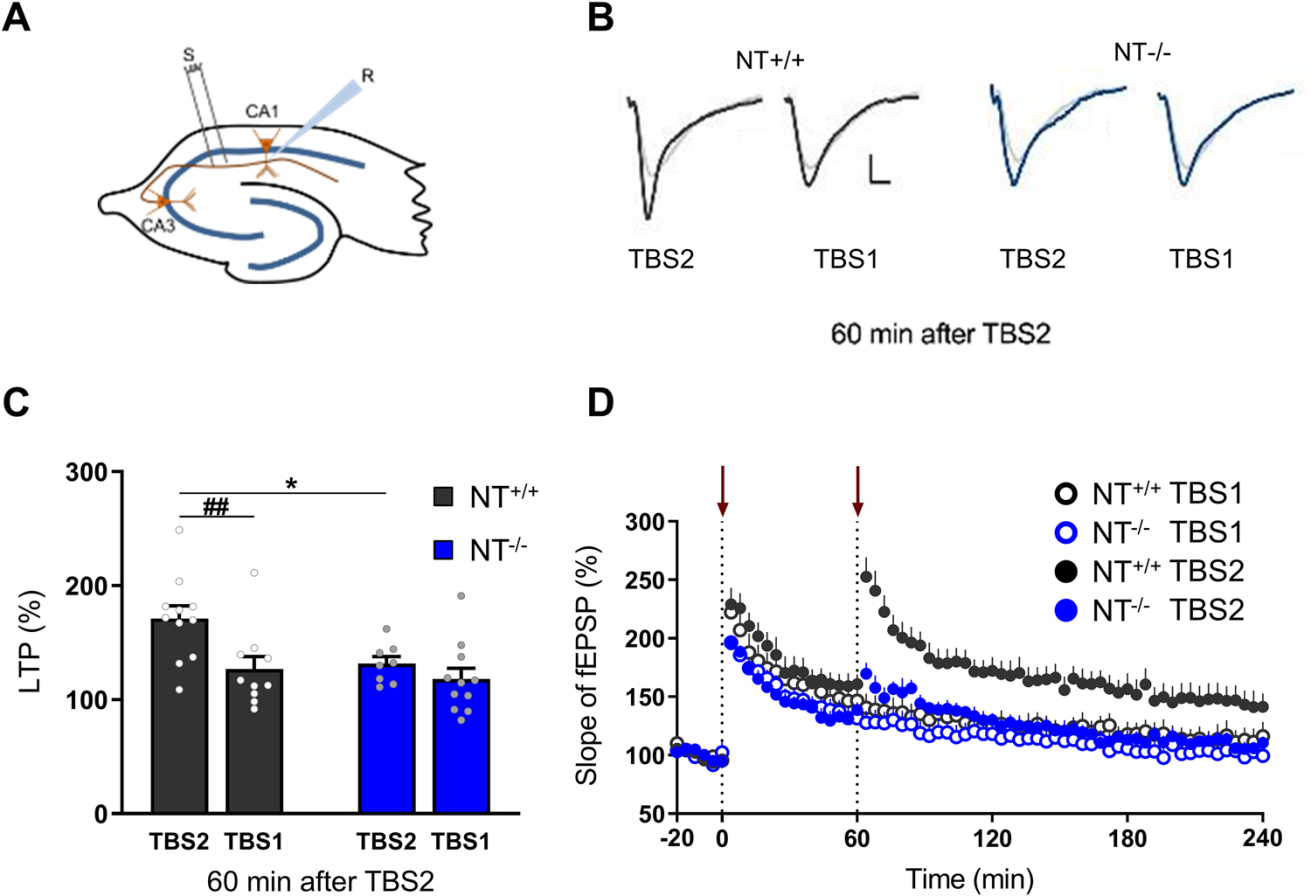
Spaced LTP in CA3-CA1 synapses in hippocampal slices from juvenile NT^−/−^ and NT^+/+^ mice. A second theta-burst stimulation train (TBS2) does not produce additional potentiation in NT^−/−^ mice. **A** A schematic representation of hippocampal slices illustrating the position of both stimulating (positioned in the Schaffer collaterals) and recording (placed among apical dendrites of CA1 pyramidal cells) electrodes. **B** Representative field excitatory postsynaptic potentials (fEPSPs) from NT^+/+^ (dark gray) and NT^−/−^ (blue) slices, which were recorded during baseline (light gray) and 2 h after TBS1 in hippocampal slices that received either 1 × TBS (TBS1) or 2 × TBS (TBS2). Scale bars, 0.5 mV/2 ms. **C** A bar plot summarizing the mean LTP levels 120 min after TBS1 (60 min after TBS2). **D** Time-courses of the slope of fEPSPs show impaired spaced LTP in NT^−/−^ mice. Arrows show time points when the first and second TBSs were applied. All data are shown as the mean ± SEM. The numbers of tested NT^+/+^ and NT^−/−^ slices/mice for each group were as follows: 11/6 (NT^+/+^, 2 × TBS), 10/6 (NT^+/+^, 1 × TBS), 8/6 (NT^−/−^, 2 × TBS) and 11/6 (NT^−/−^, 1 × TBS). Two-way ANOVA and two-way repeated-measures ANOVA with Holm–Šidák post hoc test were applied. * *P* < 0.05, significant differences in LTP between genotypes. ^##^*P* < 0.01, significant difference between LTP induced by 1 × and 2 × TBS in wild-type mice

First, we investigated the importance of NT-dependent spinogenesis for functional long-term synaptic plasticity underlying learning and memory. We used a “spaced” protocol for the induction of LTP and applied two 1-h spaced TBSs [16]. The first TBS was supposed to promote filopodia generation, while the second TBS may convert silent synapses into functional synapses by recruitment of AMPA receptors. As NT is expressed in the hippocampus, we tested spaced LTP in NT^+/+^ and NT^−/−^ juvenile mice at CA3-CA1 synapses (Fig. 1A).

We found that after induction of LTP by one TBS, the levels of potentiation were similar in both genotypes (*P*_genotype_ = 0.383, *F*_(1,19)_ = 0.798; *P*_genotype x time_ = 1.000, *F*_(717, 13623)_ = 0.610; Fig. 1B–D). Application of the second TBS (TBS2) further increased the level of LTP in NT^+/+^ but not in NT^−/−^ mice (*P*_genotype_ = 0.010, *F*_(1,17)_ = 8.280; *P*_genotype x time_ < 0.001, *F*_(537,9129)_ = 2.902; Fig. 1C, D). Overall, these results indicate that juvenile mice lacking NT show no additional potentiation after TBS2, unlike control mice. These observations suggest that a new population of synapses induced by the NT-agrin signaling pathway by TBS1 could be potentiated by TBS2.

### Role of neurotrypsin in contextual fear conditioning in juvenile mice

**Fig. 2:**
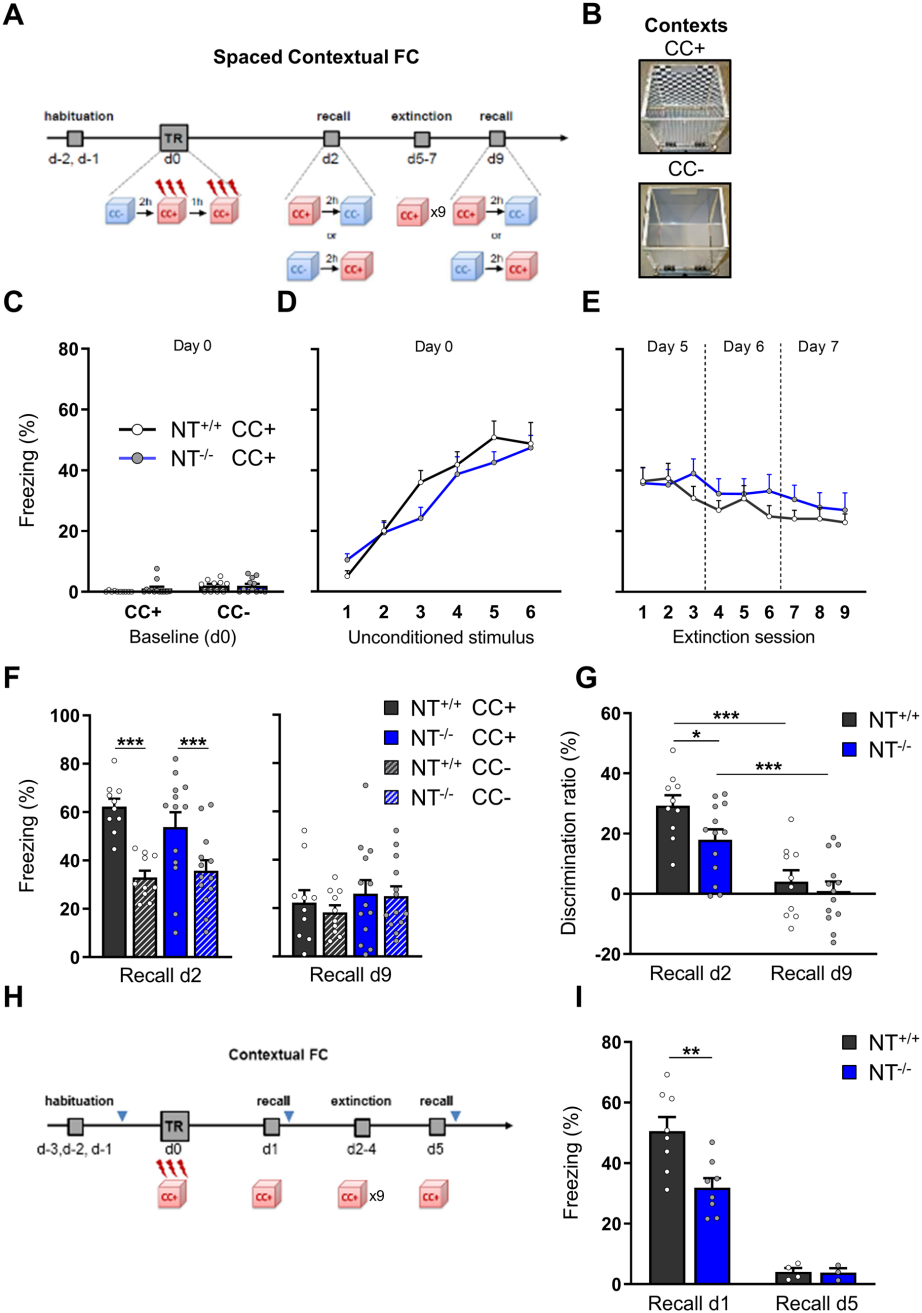
Contextual fear conditioning and extinction in juvenile NT^−/−^ and NT^+/+^ mice. NT^−/−^ mice were impaired in context discrimination but not in fear memory extinction. **A** The schematic and timeline of spaced CFC. **B** Conditioned context (CC +) and neutral context (CC-). **C** Fraction of freezing in both contexts before fear conditioning (baseline). **D** Fraction of freezing in CC + after footshocks used as unconditioned stimuli. **E** Fraction of freezing during the extinction sessions. **F** Fraction of freezing during retention of contextual fear memory revealed that both genotypes were able to distinguish between CC + and CC- at recall on d2 and both genotypes showed extinction of fear memory on d9. **G** Analysis of the discrimination ratio revealed that the ability of NT^−/−^ mice to discriminate CC + from CC- was weaker than in NT^+/+^ mice in the first recall session; after extinction, fear memory was extinguished independently of genotype. **H** The scheme and timeline of the non-spaced contextual CFC. Blue arrows indicate time points used for tissue collection for spine imaging. (I) Fraction of freezing during recalls (d1) before and (d5) after extinction. The data are shown as the mean ± SEM. NT^+/+^, *n* = 10; NT^−/−^, *n* = 13 in spaced CFC. NT^+/+^, *n* = 8; NT^−/−^, *n* = 8 in non-spaced CFC. *P < 0.05, **P < 0.01, ***P < 0.001. Two-way repeated-measures ANOVA with Holm–Šidák post hoc test was applied in C, D, E, F, and G; non-paired t test was used for the subpanel I to compare the discrimination ratios between genotypes

Previous experiments have implicated NT in hippocampal plasticity [7]. To match the spaced LTP protocol at the behavioral level, we designed a protocol of spaced contextual fear conditioning (CFC) and extinction in which six footshocks were applied in the conditioned context (CC +), divided into two learning sessions (3x + 3x) with a 1-h-interval delay between both sessions (Fig. 2A). To evaluate fear memory, we measured the freezing response (total freezing time divided by trial duration) in the conditioned context (CC + , Fig. 2B) and the neutral context (CC–, Fig. 2B) on day 2 (recall d2). To examine memory extinction, we analyzed freezing levels at day 9 (recall d9) after 9 × extinction sessions in CC + to eliminate conditioned fear. Monitoring the freezing response in the CC– context allowed us to measure the mice's ability to differentiate between both contexts.

The results indicated that NT deficiency did not affect the level of spontaneous freezing before CFC at day 0 (training) in either the CC– or CC + (*P*_genotype_ = 0.405, *F*_(1,21)_ = 0.720; *P*_genotype x context_ = 0.417, *F*_(1,20)_ = 0.688; Fig. 2C). This freezing level shown by juvenile mice before CFC was typical for the exploration of novel environments in mice. Moreover, we assessed anxiety levels and general locomotor activity in the open field test. The results demonstrate that NT deficiency did not alter general locomotor activity (*P* = 0.540; NT^−/−^: 14.4 ± 1.1 m *vs.* NT^+/+^: 15.3 ± 1.0 m; Fig. S1A), speed or immobility time (Fig. S1B, C), or anxiety levels (*P* = 0.479; NT^−/−^: 34.2 ± 3.9% *vs.* NT^+/+^: 30.6 ± 3.0%; Fig. S1D, E). Additionally, the levels of freezing immediately after unconditioned stimuli were not different between genotypes (*P* _genotype_ = 0.359, *F*_(1,21)_ = 0.880, *P*_genotype x stimulus_ = 0.250, *F*_(5,105)_ = 1.347; Fig. 2D).

Analysis of memory recall revealed that both groups of juvenile mice were able to distinguish between the CC + and CC- contexts on d2 (two-way repeated-measures ANOVA: *P*_context_ < 0.0001, *F*_(1,21)_ = 92.929; *P*_genotype_ = 0.651, *F*_(1,21)_ = 0.210; *P*_time x genotype_ = 0.031, *F*_(1,21)_ = 5.323; *P* < 0.001 for both NT^+/+^ and NT^−/−^ mice, respectively, Holm–Sidak post hoc test; Fig. 2F left). During extinction sessions from day 5 to day 7, the freezing time in CC + of both groups gradually reduced (*P*_session_ < 0.001, *F*_(8,168)_ = 7.13), but there was not statistical difference detected between genotypes (*P* _genotype_ = 0.469, *F*_(1,21)_ = 0.545; *P* _genotype x session_ = 0.245, *F*_(8,168)_ = 1.303; Fig. 2E). The fear response reduced to similar levels in all genotypes (*P* _genotype_ = 0.416, *F*_(1,21)_ = 0.688) and contexts (*P* _context_ = 0.312, *F*_(1,21)_ = 1.074) after the fear extinction protocol at recall on d9 (Fig. 2F right). However, juvenile NT^−/−^ mice appeared to be less efficient in discriminating between contexts during fear memory retrieval on day 2 (*P* = 0.026, Holm–Sidak post hoc test; Fig. 2G). To test whether impaired context discrimination was due to impaired visual function in NT^−/−^ mice, we subjected these mice to the novel object recognition test (NORT) that critically depends on visual discrimination. As NT^−/−^ mice stayed longer near novel rather than familiar objects in NORT (73.6 ± 13.6 s *vs.* 39.9 ± 12.5 s, *P* = 0.0073; Fig. S1G), similar to NT^+/+^ mice (65.7 ± 8.6 s *vs.* 38.8 ± 6.8 s, *P* = 0.00007; Fig. S1G left), it appeared that they have normal visual function and form object recognition memory normally (discrimination ratio: NT^−/−^: 42.1 ± 7.8% *vs.* NT^+/+^: 42.8 ± 4.6%, *P* = 0.937; Fig. S1H).

In these experiments, we used a strong CFC protocol, which resulted in only a mild difference between genotypes in the discrimination between contexts. To clarify whether this was due to a ceiling/saturation of CFC in both genotypes, we designed a milder CFC protocol with 3 × footshocks and single-context testing (Fig. 2H). This second CFC protocol was designed without spaced learning sessions; however, several studies suggest that sleep plays an active role in the replay of information and memory consolidation [18–20]. In this sense, acquisition followed by consolidation can be viewed as spaced stimulation. Importantly, the freezing time in the conditioned context, CC + , was significantly less 24 h after conditioning in the juvenile NT^−/−^ mice (31.9 ± 3.2% vs. 50.5 ± 4.7%; *P* = 0.005; Fig. 2I), suggesting that NT deficiency impairs the formation and/or retrieval of contextual fear memory. As in spaced CFC, both genotypes similarly decreased their freezing levels after the fear extinction protocol, indicating that the extinction of contextual fear memory was not altered by NT deficiency (Fig. 2I).

### Neurotrypsin is important for social interaction in juvenile mice

Next, we investigated social behavior in NT^−/−^ mice. Alterations in social behavior are symptoms of several neuropsychiatric and neurological diseases. In particular, mental retardation is generally accompanied by a functional deficit in adaptive behavior, such as social skills and communication [21].

**Fig. 3:**
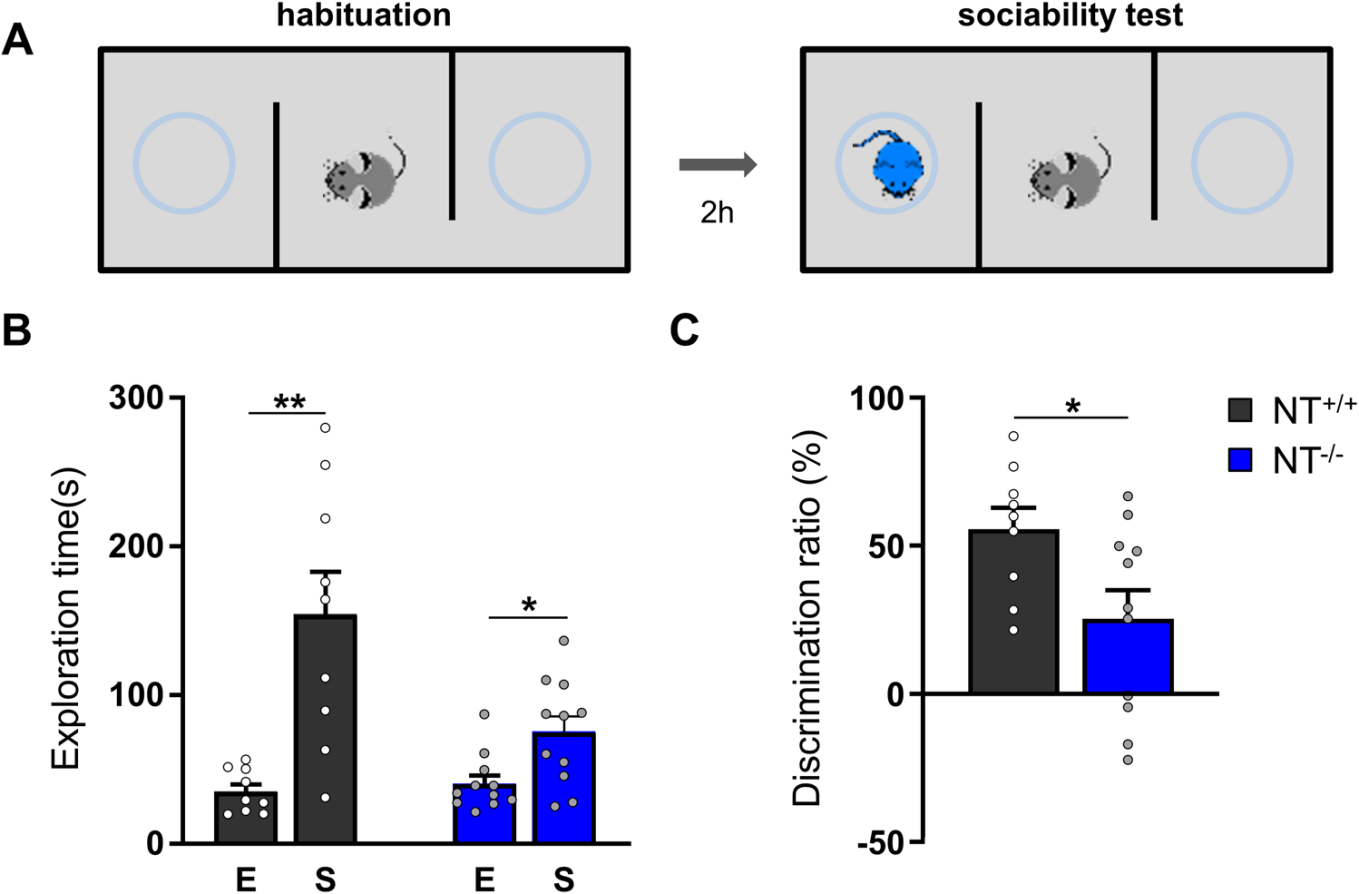
Juvenile NT^−/−^ mice showed less sociability than their NT^+/+^ littermates in the three-chamber sociability test. **A** The timeline and schematic representation of the experimental setup. **B** The total exploration duration toward the “empty box” (E) and the “stimulus mouse” (S) during the sociability test showed that both NT^+/+^ and NT^−/−^ mice spent different time in E vs. S compartments (*P < 0.05, **P < 0.01, paired t test) but NT^−/−^ mice showed less preference to the stimulus mouse. **C** The discrimination index between the E and the S compartments was significantly different between the two groups of mice: *P < 0.05, non-paired t test. The data are shown as the mean ± SEM. NT^+/+^: n = 9; NT^−/−^: n = 11

To evaluate sociability, we performed a three-chamber sociability test (Fig. 3A). We observed that juvenile NT^−/−^ mice spent less time near the box with the stimulus mouse inside (74.9 ± 10.8 s *vs.* 40.2 ± 5.7 s, *P* = 0.017; Fig. 3B) compared to their control NT^+/+^ littermates (153.9 ± 28.9 s vs. 34.9 ± 4.9 s, *P* = 0.0029; Fig. 3B). Also, the NT^−/−^ mice showed less preference for the stimulus mouse. This result was confirmed by the analysis of the discrimination ratio to exclude the individual variability in total exploration time (NT^−/−^: 25.4 ± 9.6% *vs.* NT^+/+^: 55.5 ± 7.3%, *P* = 0.0267, t test; Fig. 3C).

### Age-persistent and new behavioral defects in aged neurotrypsin-deficient mice

Next, we asked if impaired CFC and sociability in juvenile NT^−/−^ mice would persist during maturation and aging and if additional defects could appear. Hence, we evaluated the behavioral phenotype of 1- to 2-year age-matched NT^−/−^ and NT^+/+^ mice in a battery of behavioral tests (Fig. S2A).

In the open field test, the total distance traveled by aged NT^−/−^ mice was not significantly different compared with NT^+/+^ littermates (*p* = 0.343, 32.3 ± 1.6 m *vs.* 36.4 ± 3.8 m; Fig. S2B). There was also no difference between genotypes in cumulative time spent in the central (*P* = 0.285, NT^−/−^: 111.2 ± 14.6 s; NT^+/+^: 134.7 ± 15.5 s) and peripheral areas (*P* = 0.284, NT^−/−^: 488.9 ± 14.6 s; NT^+/+^: 465.3 ± 15.5 s) (Fig. S2C, D). Due to a slight trend for NT^−/−^ mice to spend less time in the central area, the elevated plus maze was additionally used to verify whether these mice were more anxious. However, NT^+/+^ and NT^−/−^ littermates spent similar amounts of time in both open arms (*P* = 0.827, 112.4 ± 13.8 *vs.* 108.5 ± 11.7 s) and enclosed arms (*P* = 0.855, 419.9 ± 16.0 s *vs.* 423.7 ± 12.7 s). There was no significant difference in discrimination ratio for the time spent in the arms (*P* = 0.820; NT^−/−^: 57.6 ± 5.3%*;* NT^+/+^: 59.2 ± 4.5%; Fig. S2E-G). These results suggest that NT deficiency influences neither locomotor activity nor anxiety status.

Next, we performed a series of cognitive tests in which animals with a normal ability to memorize objects/animals during the encoding phase should spend more time exploring a new object/animal or familiar objects in a new location during the test phase in the novel object recognition test or novel object location test, respectively. In the novel object recognition test, aged NT^+/+^ animals indeed spent more time exploring a novel object than a familiar one (*P* = 0.0003; 31.5 ± 2.5 s *vs.* 14.8 ± 1.5 s), whereas aged NT^−/−^ mice spent similar amounts of time exploring novel and familiar objects (*P* = 0.460; 23.6 ± 2.2 *vs.* 20.4 ± 2.5 s; Fig. S3A, subpanel 1). There was a significant difference in the discrimination ratio between genotypes (*P* = 0.024; NT^−/−^: 8.0% ± 9.1%*;* NT^+/+^: 35.1% ± 6.6%; Fig. S3A, subpanel 2). In the novel object location task, aged NT^+/+^ mice spent similar time exploring an object with a changed spatial position and the one which was unchanged during the retrieval phase (*P* = 0.149; 22.3 ± 2.7 *vs.* 19.6 ± 2.7 s), also aged NT^−/−^ mice spent the same time exploring both objects (*P* = 0.474; 23.9 ± 1.0 s *vs.* 26.3 ± 2.7 s; Fig. S3B, subpanel 1). The difference in discrimination ratio between genotypes was not statistically significant (*P* = 0.162, non-paired t test; Fig. S3B, subpanel 2).

To test the ability of NT^−/−^ mice to memorize the temporal sequence of events, we performed a temporal order recognition task. However, both NT^−/−^ and NT^+/+^ aged mice failed to spend more time exploring the object less recently shown than they spent exploring the other object (*p* = 0.346 and 0.799, respectively; Fig. S3C, subpanel 1). There was no difference in the discrimination ratio between genotypes (*P* = 0.278; NT^−/−^: -15.6% ± 8.6%, NT^+/+^: -2.0% ± 8.5%; Fig. S3C, subpanel 2). To test the capacity of aged NT^−/−^ mice to recognize and memorize other mice, we performed the social recognition test. Here, mice of both genotypes exhibited some preference for novel mice (NT^−/−^: 12.8% ± 10.0%, NT^+/+^: 18.9% ± 7.7%; Fig. S3D, subpanel 1). The exploration time that aged NT^+/+^ animals spent around novel animals tended to be higher than the time spent around familiar animals (*P* = 0.053; 53.4 ± 6.2 s *vs.* 36.1 ± 4.7 s; Fig. S3D). Thus, aged NT^−/−^ mice showed at least mild impairment in two recognition memory tasks compared to age-matched NT^+/+^ mice, indicating deficits in memory formation.

**Fig. 4:**
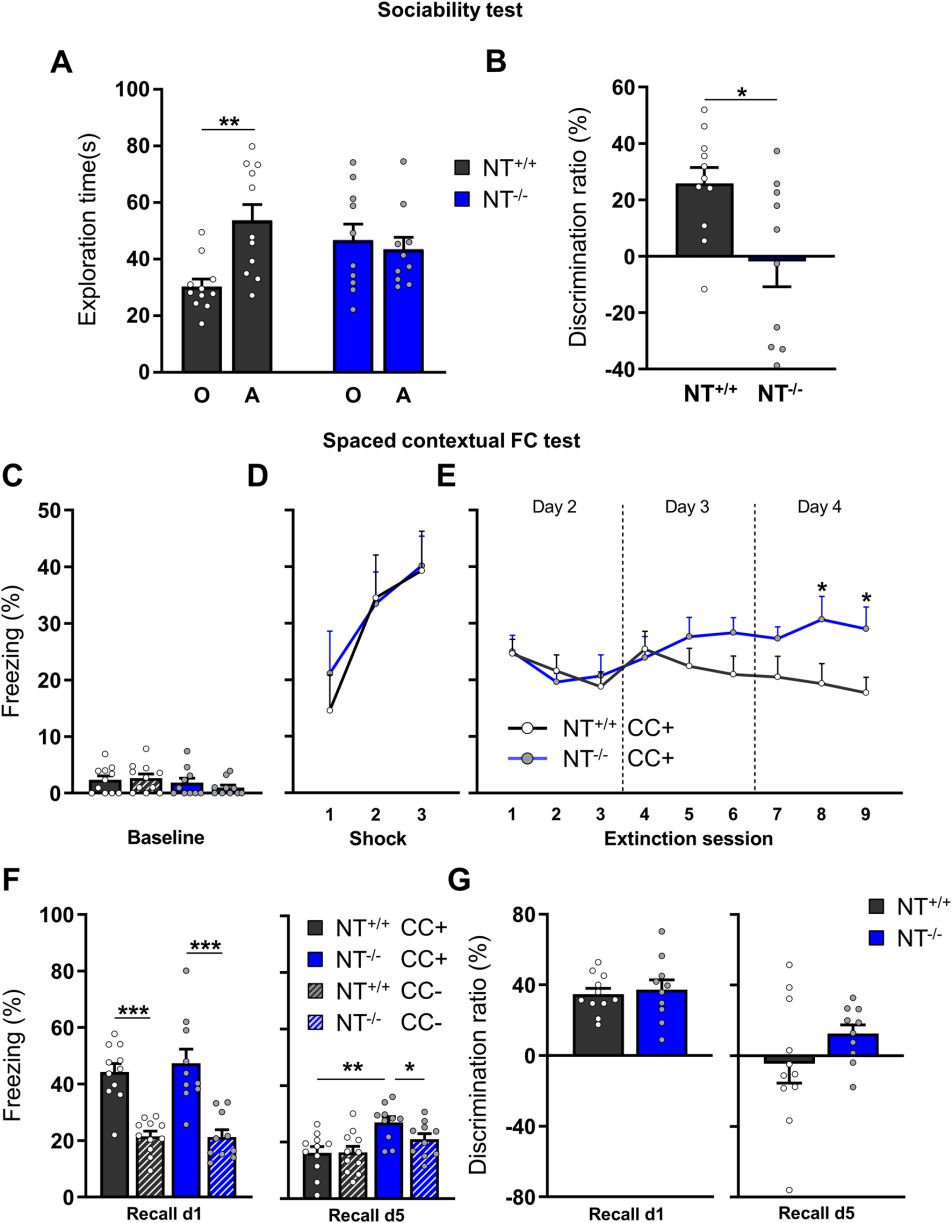
Aged NT^−/−^ mice showed highly significant deficits in sociability and fear memory extinction. **A** The total exploration duration toward the “object” [O] and the “stimulus animal” [A] during the sociability test. **B** The discrimination ratio between the “stimulus animal” and “object” was calculated as a fraction of exploration times for “stimulus animal” and “object”. **C** Fraction of freezing duration in both contexts before fear conditioning (baseline). **D** Freezing fraction in CC + after unconditioned stimuli (footshocks). **E** Freezing fraction during the extinction sessions. Percentage of freezing time in both contexts after fear conditioning on day 1 and (F, left) on day 5 (F, right). **G** Discrimination ratio calculated based on freezing time during recall sessions. The data are shown as the mean ± SEM. NT^+/+^: n = 11; NT^−/−^: n = 10. *P < 0.05, **P < 0.01, ***P < 0.001. Two-way repeated-measures ANOVA with Holm–Šidák post hoc test was applied in C, D, E, and F; paired t test was applied to compare exploration time within the same group for panel A; non-paired t test was used in B, and G to compare the discrimination ratios between genotypes

In line with data obtained in juvenile mice, aged NT^−/−^ mice failed in the sociability test, showing an almost equal level of interest in a “stimulus” mouse and a control object (*P* = 0.705; 46.6 ± 5.8 *vs.* 43.3 ± 4.4 s), whereas aged NT^+/+^ mice spent more time engaging in social communication than object exploration (*P* = 0.0027; 53.5 ± 5.8 *vs.* 43.3 ± 4.4 s; Fig. 4A). The discrimination ratio in this test differed significantly between genotypes (*P* = 0.015; KO: -1.9% ± 8.9% *vs.* NT^+/+^: 25.9% ± 5.6%; Fig. 4B).

On day 0 of CFC, both aged NT^−/−^ and NT^+/+^ littermates exhibited freezing less than 3% of the total time in the CC + and CC- before footshock (Fig. 4C). Two-way repeated-measures ANOVA revealed a significant increase in freezing time in CC + immediately after footshocks (*P*_shock times_ < 0.001, *F*_(2,38)_ = 12.083; Fig. 4D) with no difference between genotypes (*P*_genotype_ = 0.785, *F*_(1,19)_ = 0.077; *P*_shock times x genotype_ = 0.700, *F*_(2,38)_ = 0.360; Fig. 4D). Thus, aged NT^−/−^ mice perceived the unconditioned stimulus and had the same ability as aged NT^+/+^ mice to form and express fear memory. On day 1, fear memory was evaluated in a recall session. As shown in Fig. 4F (left), animals spent more time freezing in the CC + than in the CC- (*P*_context_ < 0.001, *F*_(1,19)_ = 69.398), as revealed by the Holm–Sidak post hoc test within the NT^−/−^ (*P* < 0.001, 47.4% ± 4.9% *vs*. 21.3% ± 2.6%) and NT^+/+^ groups (*P* < 0.001, 44.3% ± 3.1% *vs.* 21.5% ± 1.8%). The discrimination ratios were not different between genotypes (*P* = 0.701, NT^−/−^: 37.3% ± 5.7%; NT^+/+^: 34.8% ± 3.3%; Fig. 4G (left)). Thus, after maturation, NT^−/−^ and NT^+/+^ mice have a similar ability to discriminate contexts and recall fear memory.

From day 2 to day 4, animals experienced nine sessions (3 × each day) in the CC + conditions to induce contextual fear memory extinction. Two-way repeated-measures ANOVA revealed a statistically significant interaction between genotype and extinction sessions (*P*_test phase x genotype_ = 0.0072, *F*_(8,152)_ = 2.754; Fig. 4E); post hoc analysis indicated a significant difference between aged NT^−/−^ and NT^+/+^ mice in session 8 (*P* = 0.014, 30.7% ± 4.1% *vs.* 19.3% ± 3.5%) and session 9 (*P* = 0.014, 29.0% ± 3.9% s *vs.* 17.7 ± 2.8%). Thus, aged NT^−/−^ mice failed to exhibit contextual fear memory extinction. On day 5, another recall test was done to evaluate animals’ performance in both the CC- and CC + contexts. Two-way repeated-measures ANOVA revealed a statistically significant difference in freezing time between genotypes (*P*_genotype_ = 0.005, *F*_(1,19)_ = 9.853; Fig. 4F right). While aged NT^+/+^ mice spent an almost equal time freezing in the CC + and CC- (*P* = 0.948, 16.4% ± 2.2% *vs.* 16.2% ± 2.2%), aged NT^−/−^ mice still spent more time freezing in the CC + than in the CC- (*P* = 0.030, 26.9% ± 2.1% *vs.* 21.1% ± 1.9%). Due to the large variance, the difference in the discrimination ratio did not reach statistical significance (*P* = 0.184, 12.6% ± 5.0% *vs.* -4.5% ± 10.9%; Fig. 4G right). Nevertheless, considering all differences between genotypes on days 2–5, we conclude that aged NT^−/−^ mice (unlike aged NT^+/+^ mice) failed to exhibit contextual fear memory extinction.

### Neurotrypsin regulates spine density and morphology in juvenile mice

**Fig. 5:**
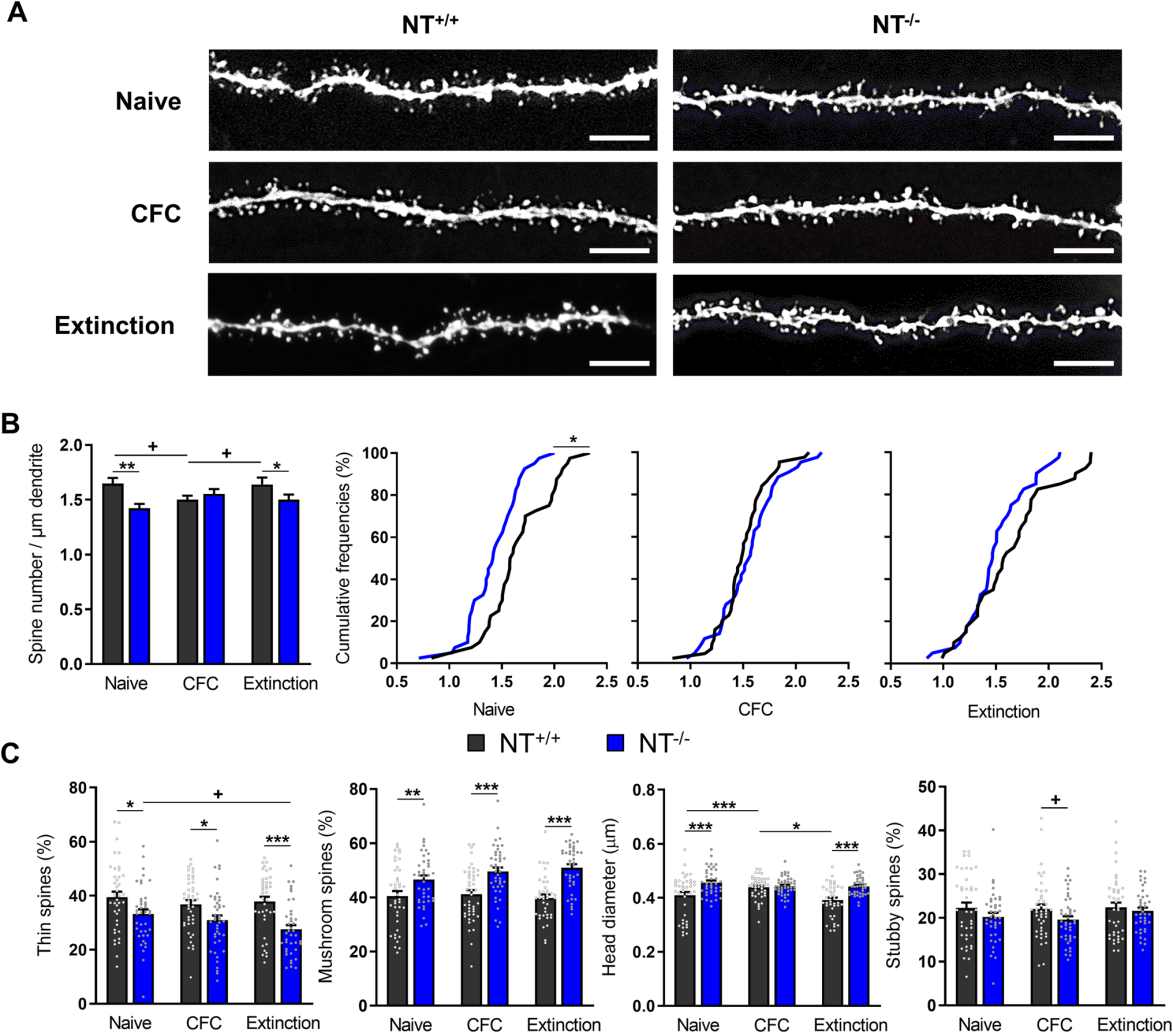
Blunted modulation in the density of dendritic spines following CFC and extinction in juvenile NT^−/−^ mice. **A** Representative images of CA1 secondary apical dendrites from NT^+/+^ mice (left column) and their NT^−/−^ littermates (right column) during different stages of CFC experiments (rows). Scale bar, 5 μm. **B** Dynamic of average and cumulative densities of spines in NT^+/+^ and NT^−/−^ mice during CFC experiment. **C** Overall spine head size and spine-type composition in NT^+/+^ and NT^−/−^ mice during CFC experiment. The numbers of analyzed NT^+/+^ and NT^−/−^ dendrites/mice for each group were as follows: 40/5 (NT^+/+^ naïve), 44/5 (NT^+/+^ CFC), 40/5 (NT^+/+^ extinction), 40/5 (NT^−/−^ naïve), 43/5 (NT^−/−^ CFC), and 40/5 (NT^−/−^ extinction). ^+^*P* < 0.1, *P < 0.05, **P < 0.01, ***P < 0.001. Two-way ANOVA with Holm–Šidák post hoc test was applied in B and C; Kolmogorov–Smirnov test was used to evaluate significance for cumulative densities (B, subpanel 2, 3 and 4)

As LTP-dependent formation of filopodia is abolished in mice lacking NT [7] and dendritic filopodia are thought to be direct precursors of new dendritic spines [22–24], we addressed the question of how NT affects spinogenesis and spine morphology in naïve conditions and upon learning. For this purpose, we crossbred NT mice with Thy1-EGFP mice, and then analyzed spine density and morphology in CA1 secondary apical dendrites (Fig. 5A) in naïve juvenile mice, 24 h after contextual fear conditioning and 24 h after fear memory extinction.

The results revealed striking differences between the two genotypes. Juvenile NT^−/−^ mice showed significantly reduced spine density in naïve and extinction conditions compared with their control NT^+/+^ littermates (two-way ANOVA: *P*_condition_ = 0.621, *F*_(2, 241)_ = 0.478; *P*_genotype_ = 0.0076, *F*_(1, 241)_ = 7.252; *P*_condition x genotype_ = 0.0111, *F*_(2, 241)_ = 4.590; *P *_*naive*_ = 0.0010, *P *_*extinction*_ = 0.0422, Holm–Sidak post hoc test; Fig. 5B, subpanel 1). Cumulative frequency curves showed that the spine density distribution shifted toward lower values in naïve NT^−/−^ mice (KS test: *p* = 0.0149, Fig. 5B, subpanel 2). However, no difference in cumulative frequency curves was found between genotypes after the acquisition or extinction of fear conditioning (Fig. 5B, C, subpanels 3,4). The absence of statistically significant differences between genotypes after extinction using the KS test is consistent with its lower power compared to ANOVA to detect a shift in Gaussian-like distributions.

Interestingly, morphological analysis determined that the percentage of thin/filopodia-like spines was significantly reduced in juvenile NT^−/−^ mice (two-way ANOVA: *P*_condition_ = 0.101, *F*_(2, 241)_ = 2.318; *P*_genotype_ < 0.001, *F*_(1, 241)_ = 28.661; *P*_condition x genotype_ = 0.370, *F*_(2, 241)_ = 0.998; *P *_*naive*_ = 0.011; *p *_*CFC*_ = 0.013, *P*_extinction_ < 0.001, Holm–Sidak post hoc test; Fig. 5C, subpanel 1), whereas the proportion of mushroom spines was higher in this genotype than in wild-type mice (two-way ANOVA: *P*_condition_ = 0.386, *F*_(2, 241)_ = 0.957; *P*_genotype_ < 0.001, *F*_(1, 241)_ = 48.813, *P*_condition x genotype_ = 0.257, *F*_(2, 241)_ = 1.365; *P *_*naive*_ = 0.005; *P*_CFC_ < 0.001, *P*_extinction_ < 0.001, Holm–Sidak post hoc test for comparisons shown in Fig. 5C, subpanel 2). In agreement with these observations, we found a statistically significant reduction in spine head size in juvenile NT^+/+^ mice (two-way ANOVA: *P*_condition_ = 0.0099, *F*_(2, 241)_ = 4.708; *P*_genotype_ < 0.001, *F*_(1, 241)_ = 26.921; *P*_condition x genotype_ = 0.005, *F*_(2, 241)_ = 5.402; *P *_*naive*_ < 0.001, *P *_*extinction*_ < 0.001, Holm–Sidak post hoc test). However, this reduction was not present after contextual fear conditioning (Fig. 5C, subpanel 3). Regarding stubby spines, no significant differences were found between genotypes and conditions (Fig. 5C, subpanel 4).

Taken together, it appeared surprising that the spine head diameter was not smaller in the control group after CFC, as in all conditions (also in CFC), the percentage of thin spines was higher in the control group and the percentage of mushroom spines was larger in the mutant group. Initially, we speculated that this could be due to mature mushroom spines in the control group being larger and more mature after CFC, thus compensating for this difference. However, we observed that the mushroom spine head size was very similar in both groups of mice (*P* = 0.286, 0.551 (0.520, 0.613) *vs*. 0.532 (0.518, 0.573); as median (quartile 1, quartile 3), Fig. S4A, B). Consequently, we measured the head diameter in the thin spines after CFC. Interestingly, we observed that the thin spines were larger and most likely more mature in NT^+/+^ mice (*P* = 0.0003, 0.2849 ± 0.006 *vs*. 0.2579 ± 0.004; Fig. S4C), suggesting that NT deficiency may specifically affect the maturation of thin/filopodia-like spines. In agreement with this, a cumulative frequency plot of spine head diameter of thin spines revealed that juvenile NT^−/−^ mice had a leftward shift in the cumulative curve, indicating a reduction in the head diameter for this spine type in the mutant group of mice (Fig. S4D).

### Neurotrypsin regulates spine morphology but not spine density in aged mice

**Fig. 6:**
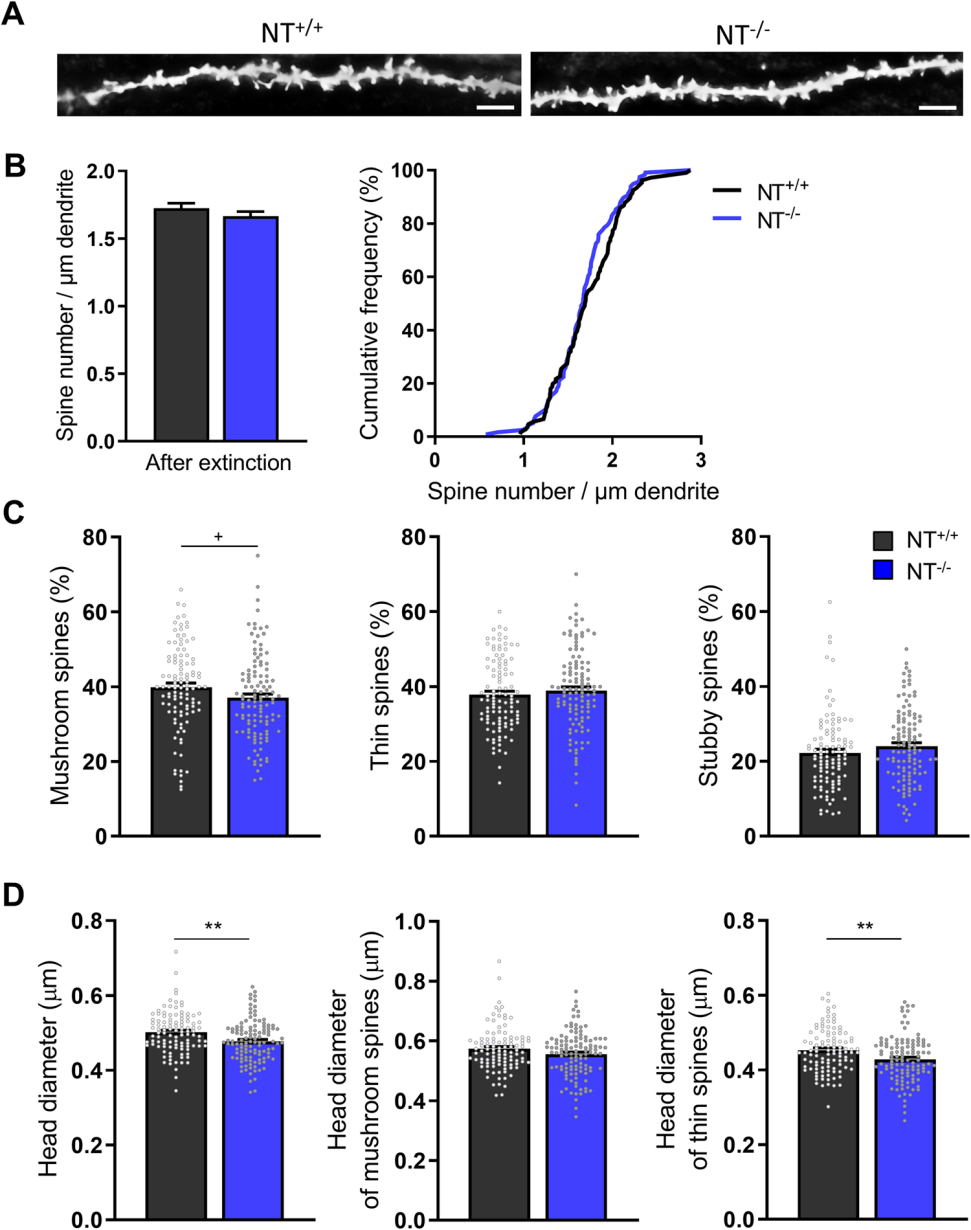
Density and morphology of dendritic spines after CFC extinction in aged NT^−/−^ and NT^+/+^ mice. **A** Representative images of CA1 secondary apical dendrites from NT^+/+^ mice (left) and their NT^−/−^ littermates (right) after CFC extinction experiments. Scale bar, 5 μm. **B** Mean values ± SEM and cumulative densities of spine density in NT^+/+^ and NT^−/−^ mice. **C** Proportions of mushroom-like, thin and stubby spines. **D** The head diameter of all, mushroom-like and thin spines. The numbers of analyzed NT^+/+^ and NT^−/−^ dendrites/mice for each group were as follows: 106/6 (NT^+/+^), 116/6 (NT^−/−^). ^+^*P* < 0.1, *P < 0.05, **P < 0.01

Since abnormalities in the spine density and morphology were observed in juvenile NT^−/−^ mice, we studied if the same trends can be observed in older mice. For this purpose, we visualized dendrites and spines using Golgi–Cox staining (Fig. 6A) in brains collected after the end of behavioral testing of aged mice (i.e., after extinction). NT^−/−^ mice turned out to have similar mean spine density as their NT^+/+^ littermates (t test: *P* = 0.256, *t*_(220)_ = 1.139; Fig. 6B, subpanel 1). Analysis of cumulative frequency curves also did not reveal a statistical difference between genotypes (KS test: *P* = 0.1586, Fig. 6B, subpanel 2).

Morphological analysis revealed a trend in the reduction of the proportion of mushroom-like spines in NT^−/−^ mice (t test: *P* = 0.0630, *t*_(220)_ = 1.869), whereas the proportions of thin and stubby spines were not significantly changed as compared to wild-type mice (thin spines, t test:* P* = 0.436, *t*_(220)_ = -0.780; stubby spines, Mann–Whitney rank sum test: *P* = 0.113, *U* = 5390.5; Fig. 6C). To match the analysis done in juvenile mice, we also analyzed the spine head width for all spines as well as separately for mushroom and thin spines. A significant reduction of all spine heads in aged NT^−/−^ mice was revealed (t test: *P* = 0.0012, *t*_(220)_ = 3.284; Fig. 6D, subpanel 1). Similar to juvenile mice, the spine head width was not reduced in mushroom spines (Mann–Whitney rank sum test: *P* = 0.152, *U* = 5463.5; Fig. 6D, subpanel 2), but was reduced in thin spines (t test: *P* = 0.0025, *t*_(220)_ = 3.063; Fig. 6D, subpanel 3).

### Neurotrypsin-dependent cleavage of agrin plays a role in regulating dendritic spine formation and synaptic growth

As agrin is the only substrate of NT identified so far and its 22 kDa cleavage fragment is critically important for activity-dependent filopodia formation [7], we addressed the question of whether agrin cleavage is responsible for the putative effects of NT deficiency. To test this hypothesis, we aimed to deliver agrin-22 in the hippocampus of juvenile NT^−/−^ mice and evaluate its effect on dendritic spines. For this purpose, we designed AAV constructs expressing agrin-22 or a control agrin-15 specifically in neurons. An N-terminal secretion signal sequence and C-terminal red fluorescent reporter protein scarlet were added to both these forms of agrin (Fig. S5A, B).

**Fig. 7:**
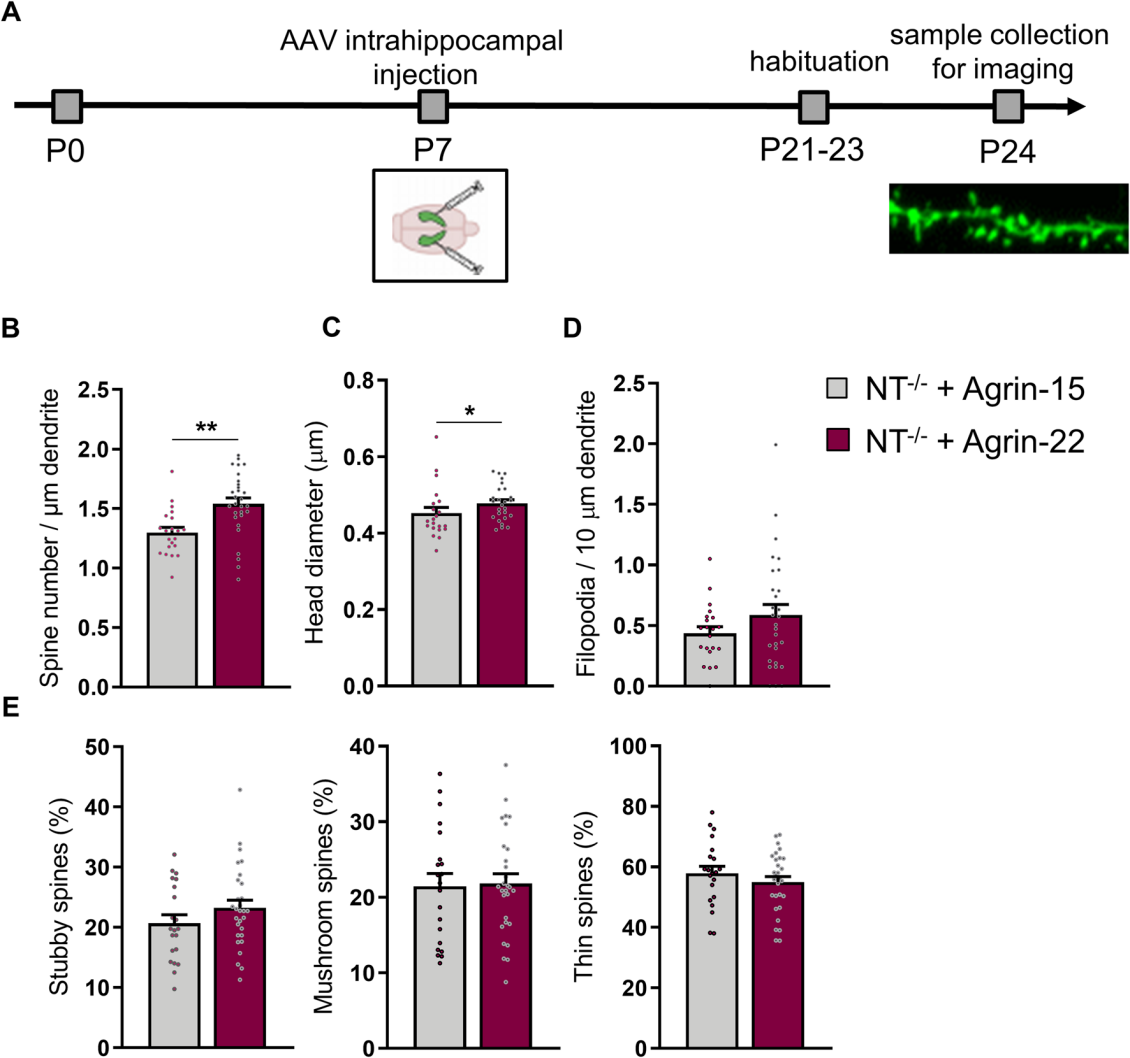
AAV-Ag22 increases the spine density in juvenile NT^−/−^ mice as compared to AAV-Ag15. **A** Schematic representation of the experiment time course. **B** Changes in spine density after injection of agrin expressing AAVs. **C** Increased spine head size in AAV-Ag22 injected mice compared to AAV-Ag15 injected mice. **D** No difference between treatments in the occurrence of filopodia. **E** Spine type composition and filopodia density in naïve NT-/- mice after AAV-Ag15 or AAV-Ag22 injection. The data are presented as the mean ± SEM values per dendrite. The numbers of analyzed dendrites/mice were 29/4 (NT^−/−^ injected with AAV-Ag22) and 21/3 (NT.^−/−^ injected with AAV-Ag15). Experimental data presented as means ± SEM. *P < 0.05, **P < 0.01, ***P < 0.001 (non-paired t test)

We injected either pAAV-Syn-Agrin22-Scarlet (AAV-Ag22) or pAAV-Syn-Agrin15-Scarlet (AAV-Ag15) at postnatal day 7 (P7) into the hippocampus of NT^−/−^ mice. Subsequently, we collected samples for spine imaging at P24 after 3 consecutive days of habituation (see Fig. 7A for a general scheme of the experiment) to follow the same protocol as that used in the previous spine analysis.

As shown in Fig. 7B, injection of AAV-Ag22 expressing virus increased spine density by 19% from 1.3 to 1.5 spines per µm (*P* = 0.001) compared with AAV-Ag15 control. In addition to its effect on spine density, injection of AAV-Ag22 in NT^−/−^ mice showed larger spine head width compared with the AAV-Ag15 treated group (*P* = 0.030; median (quartile 1, quartile 3) for NT^−/−^ + Ag22: 0.478 (0.441, 0.515) µm vs. NT^−/−^ + Ag15: 0.432 (0.412, 0.480) µm, Fig. 7C). Moreover, filopodia density was apparently slightly increased in mice injected with AAV-Ag22 (0.508 (0.199, 0.874)) compared with those injected with AAV-Ag15 (0.471 (0.298, 0.569)), but did not reach statistical significance (*P* = 0.275, Fig. 7D). The fractions of mushroom-like, thin and stubby spines were not different between groups (*P* = 0.186, Fig. 7E).

To ensure proper injections, each hippocampal slice was imaged to detect Ag22-scarlet or Ag15-scarlet expression, and only those animals with positive expression were selected for subsequent spine analysis. Both Ag22-scarlet and Ag15-scarlet labeling were distributed over neuronal somas from the CA1 *stratum pyramidale* and showed expression in the CA1 *stratum radiatum*. Distinctly, all mice injected with AAV-Ag15 exhibited a more diffuse distribution in the CA1 *stratum radiatum*, presumably because of less binding to agrin receptors and, hence, better clearance (Fig. S5C, D).

To determine whether Ag22-scarlet colocalizes with its potential neuronal receptor α3NKA (α3 Na^+^/K^+^ ATPase) (Hilgenberg et al., 2006), CA1 slices from brains injected with AAV-Ag22 were labeled with the α3NKA monoclonal antibody. Consistent with a previous in vitro study (Hilgenberg et al., 2006), we observed an extensive overlap between α3NKA and agrin-22 puncta expression in the *stratum radiatum* (Fig. S6A). To confirm that AAV-Ag22 was properly delivered and present at synaptic sites, we stained CA1 slices from brains injected with AAV-Ag22 with the excitatory presynaptic marker VGLUT1. Colocalization of scarlet-tagged Ag22 and immunostained VGLUT1 confirmed that agrin-22 was concentrated at a subset of synapses (Fig. S6B). Altogether, these observations provide evidence that virally expressed agrin-22 is present at synaptic sites in the CA1 *stratum radiatum* and co-accumulate with its physiological neuronal receptor α3NKA.

**Fig. 8:**
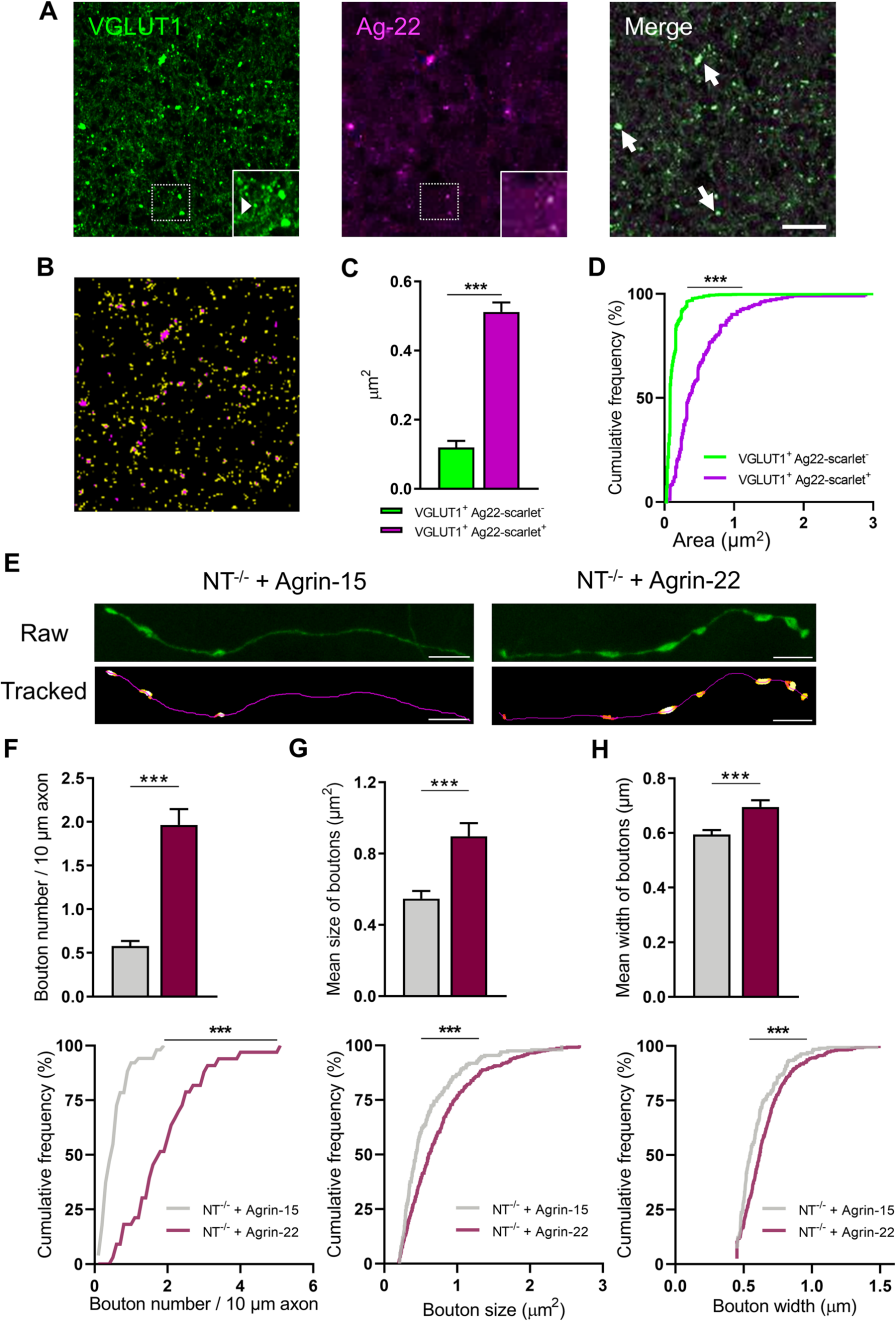
Agrin-22 colocalizes with VGLUT1 in excitatory synapses and increases axonal bouton density and size in juvenile NT^−/−^ mice. **A** Coimmunostaining of VGLUT1 and agrin-22 **B** ROIs corresponding to the VGLUT1 binary map were superimposed on the Ag22-scarlet binary map to analyze the size of both synaptic populations. **C** Comparison of agrin-22 negative (green) and agrin-22 positive (magenta) areas of VGLUT1^+^ synapses. **D** Cumulative frequency plot of the Ag22^+^ and Ag22^−^ VGLUT1^+^ presynaptic area. The data are presented as the mean ± SEM values per dendrite. The numbers of analyzed synapses/slices are 1087/3 (VGLUT1^+^ Ag22-scarlet^−^) and 214/3 (VGLUT1^+^ Ag22-scarlet^+^). **E** Typical images and traced axons and boutons used for axonal bouton analysis. **F** Increase in the bouton density after injection of agrin-22 expressing AAV, cumulative frequency curves are shown in a lower subpanel. **G** Increased bouton size in AAV-Ag22 injected mice compared to AAV-Ag15 injected mice. **H** Increases of bouton width after AAV-Ag22 treatment compared to AAV-Ag15 treated group. In **G, H**, upper subpanels present data as the mean ± SEM per axon; the cumulative frequency plots in lower subpanels represent data for all individual boutons. The numbers of boutons/axons in this analysis are 195/51 for AAV-Agrin-15-treated group and 436/33 for AAV-Agrin-22-treated group. ***P < 0.001. Non-paired t test was applied in panel C; Mann–Whitney rank sum test was applied in upper subpanels of F, G, and H; KS test was applied for comparisons of cumulative frequency distribution curves (in panel D and lower subpanels of F, G, and H)

Next, we investigated the effect of Ag22-scarlet on VGLUT1 puncta. Interestingly, we observed that VGLUT1-positive presynapses colocalizing with Ag22-scarlet were significantly larger than those without Ag22 scarlet colocalization (0.512 ± 0.027 μm^2^ *vs*. 0.120 ± 0.019 μm^2^; *P* < 0.0001; Fig. 8A–C). In agreement with this, a cumulative frequency plot of VGLUT1-immunopositive puncta area revealed that the presynapses colocalizing with Ag22-scarlet had a rightward shift in the cumulative curve, indicating an enlargement of VLGUT1 presynapses colocalizing with Ag22-scarlet (Fig. 8D). This striking observation suggests that Ag22-scarlet may induce synapse formation or aggregation of VGLUT1-positive synaptic vesicles at presynapses. Furthermore, to examine whether these large structures are composed of presynaptic but also postsynaptic specializations, we stained CA1 slices from brains injected with AAV-Ag22 with the excitatory presynaptic marker VGLUT1 and the excitatory postsynaptic marker PSD95. Interestingly, we observed complex synapses with multiple postsynaptic densities contacting agrin-22 clusters (see the magnification in the upper right corner of Fig. S7).

Finally, we investigated whether these changes in synaptic markers in AAV-Ag22-treated NT^−/−^ juvenile mice were also associated with a higher axonal bouton density and larger size of *en passant* presynaptic boutons along axons of EGFP-expressing neurons (Fig. 8E) in the same cohorts of mice as used above for the spine analysis. As shown in Fig. 8F, NT^−/−^ mice treated with AAV-Ag22 presented a higher density of axonal boutons with a median of 1.829 (quartile 1 and quartile 3: 1.294 and 2.532) boutons per 10 μm axon than AAV-Ag15 treated mice with the median of 0.493 (0.309, 0.715) boutons per 10 μm axon (Mann–Whitney rank sum test, *P* < 0.001, Fig. 8F subpanel 1). Analysis of cumulative frequency curves also revealed a difference between the treatment groups (KS test: *p* < 0.001, Fig. 8F, subpanel 2). In addition, AAV-Ag22-treated mice presented larger axonal boutons than AAV-Ag15-treated counterparts (0.613 (0.384, 0.969) μm^2^ *vs.* 0.435 (0.301, 0.723) μm^2^; Mann–Whitney rank sum test, *P* < 0.001, Fig. 8G subpanel 1). Also, analysis of cumulative frequency curves showed a difference (KS test: *P* < 0.001, Fig. 8G, subpanel 2). In terms of the bouton width, the effect of AAV-Ag22 was milder but also statistically significant compared to AAV-Ag15 (0.620 (0.524, 0.728) μm vs. AVV-Ag15*:* 0.547 (0.493, 0.651) μm); Mann–Whitney rank sum test: *P* < 0.001, Fig. 8H subpanel 1; KS test: *P* < 0.001, Fig. 8H, subpanel 2). Thus, these findings suggest that Agrin-22 may promote the formation and growth of axonal boutons.

## Discussion

Altogether, our results demonstrate the important role of NT in functional synaptic plasticity in different types of learning and memory and sociability in juvenile and mature/aged mice. Moreover, we highlight the impact of NT-dependent cleavage of agrin in regulating dendritic spine formation and morphology.

It has been previously reported that the level of theta-burst stimulation-induced LTP is normal in acute hippocampal slices from juvenile NT^−/−^ mice. One of the most striking phenotypes in these mice was the lack of formation of dendritic filopodia after induction of cLTP, which are very likely to be precursors of newly formed synapses [7]. However, the conventional LTP protocol may not be suitable to reveal an increase in synaptic transmission due to the formation of new spines, as nascent spines are mostly silent (i.e., they lack AMPA receptors) [12–14, 25]. Krámar and colleagues reported that a second TBS (TBS2) administered at least 60 min after the first TBS (TBS1) doubled the level of LTP and induced actin polymerization in more synapses compared to TBS1, supporting the idea that a new population of synapses was potentiated by TBS2 [16]. The time interval of at least 60 min was crucial to induce an additional LTP, suggesting that this time interval is necessary for the recruitment of postsynaptic density components to nascent synapses; this process can be used as a scaffold for the recruitment of NMDA and AMPA receptors. As this spaced form of LTP induced by two 1-h spaced TBSs relies on filopodia generation and their conversion into functional synapses [16], we hypothesized that it might be affected in NT^−/−^ mice. Hence, following the protocol from Krámar et al., 2012, we recorded LTP induced by two TBSs spaced by 1 h in CA3-CA1 synapses from NT^+/+^ and NT^−/−^ mouse slices. Our recordings revealed that NT^−/−^ mice show no additional potentiation after TBS2, unlike NT^+/+^ mice, suggesting that a new population of synapses induced by NT-agrin signaling could be potentiated by TBS2.

Previous studies have associated a selective impairment of LTP induced by strong stimuli at CA3-CA1 synapses with a deficit in CFC or context discrimination [26, 27]. More recent studies have also described similar combinations of impairment of context discrimination and altered LTP in the CA1 region [28, 29]. Moreover, one study that linked cognitive deficits during acute neuroinflammation also supported that the CA1 region is involved in context discrimination [30]. Both hippocampal and amygdaloid NMDA receptors are involved in the acquisition of Pavlovian fear conditioning. This research implicates NMDA receptor-dependent LTP in these brain areas in the acquisition of conditional fear. Moreover, several studies have used a correlational approach to assess the role of hippocampal LTP in contextual fear conditioning, suggesting that hippocampal NMDA receptor-dependent LTP is required in encoding contextual representations [31]. Interestingly, NT activation is an NMDA receptor-dependent process [7]. Hence, this prompted the question of whether NT may play a role in the acquisition of contextual fear memory.

Our “spaced” contextual fear conditioning protocol revealed that NT deficiency did not impair the retrieval of contextual memory during memory recall at d2 (48 h after the training session). However, NT^−/−^ mice appeared to be less efficient in discriminating between contexts during the retrieval of fear memory. Combined results indicate that there may be significant redundancy in the neuroanatomical regions and compensatory mechanisms mediating fear conditioning [32]. This is not surprising, as the ability to store fear memories from frightening experiences accurately and reliably is essential to survive in an ever-changing and potentially dangerous environment. This may partly justify why the loss of NT is not sufficient to result in an impairment of encoding and/or recall of fear mnemonic processes. Another possibility to consider is the generalization effect of the context. The generalization of fear conditioning is manifested as a loss of stimulus specificity and as the emotional sensitization of associative components of memory; the generalization of fear conditioning thus leads to diminished signal discrimination and generalized anxiety [33, 34]. Therefore, the more the signal is generalized, the less discrimination occurs. Previous findings indicate that fear generalization is broadly tuned and sensitive to the intensity of unconditioned stimuli [35]. Indeed, it was shown that increasing footshock intensity [35, 36] and the number of footshocks [37] led to context memory generalization. Consequently, we performed a milder contextual fear conditioning protocol with one single context. The results obtained indicate that the NT^−/−^ mice freeze significantly less in the conditioned context 24 h after conditioning, suggesting that the NT null mutation impairs the formation or retrieval of contextual fear memory. Importantly, NT deficiency affected neither the level of freezing before fear conditioning (normal freezing level during fully unconstrained exploration of a novel environment is 5–10% in mice) nor the significant elevation of freezing between three unconditioned stimuli, indicating that differences between genotypes at the memory recall session are not due to distinct perception of the aversive stimulus or higher basal anxiety levels. On the other hand, both genotypes similarly reduced their freezing levels after fear memory extinction, demonstrating that the extinction of contextual fear memory was not altered by NT deficiency in juvenile mice.

Although impaired spaced LTP in NT^−/−^ mice highlighted NT as a potentially important factor underlying the efficacy of spaced learning, our behavioral and spine analysis data suggest a more general role of NT in both spaced and non-spaced learning. We argue that this is possibly related to the replay of acquired information during sleep, which essentially mimics the spaced learning protocol and may drive the maturation of learning-induced filopodia/immature spines independently of the number of learning episodes. This concept is in line with our data suggesting the importance of NT in both generation and survival/maturation of filopodia/thin spines, as discussed below.

Blaeser and colleagues [38] presented a study in a mouse with a mutation in the CaM kinase isoform (CaMKK), an upstream component of the Ca^2+^/calmodulin kinase (CaMK) cascade that has been implicated in neuronal gene transcription, synaptic plasticity, and long-term memory consolidation. Interestingly, they found normal long-term spatial memory formation and cued fear conditioning in CaMKK mutants but impaired contextual fear conditioning. In addition, CaMKK mutants exhibited impaired activation of the downstream kinase CaMKIV/Gr and its substrate, the transcription factor cyclic AMP-responsive element-binding protein (CREB), upon fear conditioning. Similar to our juvenile NT knockout mice, these mutants exhibited normal levels of anxiety-like behavior and no deficit at all in the extinction of the freezing response [38]. Interestingly, it was previously described that activation of the agrin receptor in neurons resulted in a tyrosine kinase-dependent increase in intracellular Ca^2+^ that engages both CaMKII and MAPK signaling pathways [39]. Moreover, CREB phosphorylation was markedly decreased in the neurons of NT^−/−^ mice after a spatial learning task and social interaction compared to hippocampal neurons of their NT^+/+^ littermates [40], suggesting that the mechanisms downstream of the agrin receptor in neurons may be involved in this signaling pathway.

Alterations in social behavior are symptoms of several neuropsychiatric and neurological diseases. In particular, mental retardation is generally accompanied by a functional deficit in adaptive behaviors, such as using social skills and engaging in communication [21] Social interaction was tested using the three-chamber sociability test, a widely used test paradigm to quantitatively measure this behavior [41]. We found that although juvenile NT^−/−^ and NT^+/+^ mice stayed near the cage with the stimulus mouse significantly longer than they stayed near the empty cage, NT^−/−^ mice have a remarkably lower preference and, hence, lower sociability levels compared with their control wild-type littermates. Older NT^−/−^ mice showed no preference for animated objects. This outcome is different from the results of a previous study in adult (14–20-week-old) NT knockout mice in which both NT^−/−^ and NT^+/+^ mice sniffed the stimulus mouse for significantly longer equal time intervals than they sniffed the empty cage [40]. Surprisingly, although normal in terms of sociability and social memory, adult NT knockout mice showed markedly reduced CREB phosphorylation in hippocampal CA1 neurons after social interaction compared to their wild-type littermates [40]. However, CREB analysis was not performed in that work in the same animals that underwent the three-chamber sociability test. Mice that were tested for behavioral analysis were housed in single cages at least 1 week before starting the task. On the other hand, brain slices for CREB analysis came from animals that were group-housed at least 2 weeks before the test. In addition, Mitsui and colleagues used a different NT knockout model for their experiments, in which exon 1 of the PRSS12 gene (the gene encoding NT) was replaced [40], while in our NT knockout mouse model, part of exons 10 and 11 was replaced, resulting in a truncated NT gene lacking the region encoding the proteolytic domain [8]. In addition, the age difference may be essential, resembling the age-dependent phenotype of mice deficient in the cell adhesion molecule CHL1, which showed impaired LTP in juvenile mice, normal LTP in 2-month-old adult mice and impaired LTP at the age of 9 months [42].

A direct comparison of phenotypes of juvenile and aged NT^−/−^ mice also revealed several differences in their performance: 1) in fear conditioning, which was impaired in juvenile mice but normal in aged ones; 2) in fear extinction, which was normal in juvenile and impaired in aged mice; and 3) in the novel object recognition test outcomes, which were also normal in juvenile and impaired in aged mice (Table S1). Additionally, a previous analysis of adult NT^−/−^ mice did not reveal significant deficits in the Morris water maze, passive avoidance, or Y-maze tests [40]. Thus, NT deficiency shows a complex pattern of interaction with age, so some defects could be compensated for in young mice but become detectable with aging. Of note, plasticity mechanisms can undergo profound changes during aging, as has been shown, e.g., for ocular dominance plasticity [43]. This results in sensitive or critical periods of development that affect maturation of the sensory system but also fear learning and memory [44] and may account for the differential outcome of behavioral experiments with NT^−/−^ mice between the ages.

As LTP-dependent formation of filopodia appeared to be abolished in mice lacking NT [7] and dendritic filopodia are thought to be direct precursors of new dendritic spines [22–24], we hypothesized that NT-dependent mechanisms may affect spinogenesis and/or spine morphology. As NT is released in an activity-dependent manner [6], we investigated dendritic spines in both naïve mice and mice after they performed learning tasks. To assess learning-induced spine formation, we selected the contextual fear conditioning task for two reasons. On the one hand, we found that NT^−/−^ mice were impaired in the formation or retrieval of CFC, as they have significantly lower freezing levels at memory recall. On the other hand, several studies have provided evidence that FC leads to changes in spine density and/or morphology, which makes that paradigm suitable for our purpose [45–51].

We observed significantly reduced spine density in CA1 secondary apical dendrites under naïve conditions in juvenile NT^−/−^ mice compared to their control wild-type littermates. This is in line with a previous study describing that a different knockout model of NT had reduced spine density in neurons from the CA1 region of the hippocampus but not from the cingulate cortex [40]. Contrary to our expectations, no differences in spine density after the acquisition or extinction of CFC (learning-dependent spinogenesis) were detected between genotypes. However, it seemed that in wild-type mice, there was a tendency to reduce spine density after the acquisition of fear memory and to increase spine density again after extinction (going back to a similar scenario like that in naïve conditions). This is in line with a study that revealed that fear conditioning increased the rate of spine elimination, whereas fear extinction increased the rate of spine formation [49]. Similarly, another study reported a significant decrease in total spine density after CFC relative to home-caged animals that did not undergo CFC [52]. This is consistent with a documented role of stress in decreasing spine density [53, 54]. In contrast, some other studies have reported no changes in spine density after CFC [47, 48] or an increase in spines after CFC [45, 46, 51]. The discrepancy between those studies might rely on the timing of sample collection for spine counting.

In summary, no spine changes were observed in juvenile NT^−/−^ mice after learning, while spine density was modulated by CFC and extinction in wild-type mice. Interestingly, the morphological analysis determined that the percentage of thin/filopodia-like spines was significantly reduced in NT^−/−^ mice in all conditions, whereas the proportion of mushroom spines was higher in this genotype. Regarding stubby spines, no significant differences were found between the two groups. Thin spines are highly dynamic protrusions that can grow, shrink, or change into other spine subtypes [55]. In contrast, stubby and mushroom spines are less dynamic and relatively stable [56, 57]. Thus, the higher percentage of thin spines in the wild-type mice likely reflects the morphological shift of spines that concurrently fulfill the need for available synaptic sites for structural plasticity.

In agreement with the observed higher proportion of thin spines in juvenile wild-type mice, we found a statistically significant reduction in spine head size in this group of mice. However, this reduction was not present after CFC. We initially thought that this enlargement of the spine head size after CFC may be due to a difference in the mushroom spines, which might be larger and more mature in the control group after they undergo CFC and hence allow them to compensate for this difference. Accordingly, we measured the head diameter in the mushroom spines that were automatically detected by the software used for spine analysis. Surprisingly, we found that mushroom spine head sizes were similar in both groups of mice. Consequently, we evaluated the head diameter in the thin spines after CFC. Strikingly, we observed that the thin spines were larger and most likely more mature in juvenile wild-type than in NT^−/−^ mice, suggesting that NT deficiency may specifically affect the maturation of thin/filopodia-like spines. Interestingly, the enlargement of nascent spines was revealed to be tightly coupled to the formation and maturation of glutamatergic synapses [58].

There was an increase in the fraction of mushroom spines concomitant with a decrease in thin/filopodial spines in juvenile NT^−/−^ mice. These data are consistent with the idea that filopodia, and hence, thin spines are less often generated in the absence of neurotrypsin/agrin-22, potentially reducing destabilization of mushroom-like spines mediated by agrin-22-induced local membrane depolarization. Additionally, agrin-22 may assist thin spines to adopt a mushroom phenotype by promoting the accumulation of synaptic proteins, which is consistent with data shown in Figs. 7 and S7.

Analysis of dendritic spines in aged mice did not reveal a reduction in the spine density in NT^−/−^ mice as in their juvenile counterparts. The overall fraction of mushroom, stubby, and thin spines was normal after extinction in contrast to the reduced fraction of thin spines in juvenile NT^−/−^ mice. Regarding the head size, the width in thin spines decreased in NT^−/−^ mice at both juvenile and matured ages; the width of mushroom spines was not largely changed at both ages. These new data suggest the specific role of neurotrypsin in filopodia/thin spine formation in juvenile mice and a more general role of NT in maturation of thin spines. The relatively mild interaction between learning and genotype and potential role of NT in different aspects of synaptic remodeling suggest that for future studies, it would be highly beneficial to use longitudinal two-photon imaging in awake mice and directly resolve learning-induced generation and maturation of dendritic spines as a function of NT activity.

As agrin is the only substrate of NT identified so far and is critically important for activity-dependent filopodia formation [7], we examined whether agrin cleavage may be responsible for the observed spine loss in NT^−/−^ mice. Interestingly, the previous research described that the transmembrane isoform of agrin regulates dendritic filopodia [59, 60] and synapse formation [59] in rat mature hippocampal neurons. Another study on agrin function in the CNS indicated that clustering of agrin using anti-agrin antibodies stimulated filopodia formation in mouse hippocampal neuronal culture [61]. We investigated whether the expression of agrin-22 could significantly increase spine density in NT^−/−^ mice as compared to expression of agrin-15. Injection of AAV-Ag22 also increased the spine head size. Still, the experiments with forced overexpression of agrin-22 cannot fully mimic the Hebbian mechanism of agrin-22 generation, which presumably involves synchronization of pre- and postsynaptic processes [7]. Thus, the viral expression of agrin-22 is not expected to fully match endogenous agrin-22 expression in terms of timing and expression levels, and hence, only some physiological effects of agrin-22 are observed after its viral overexpression. Viral agrin-22 may increase the probability of spine generation but has a lesser impact in regulating downstream processes as compared to the Hebbian process of agrin-22 generation, which may additionally mobilize pre- and postsynaptic machineries at agrin-22 generation sites. This might explain the observed increase in spine density after agrin-22 expression but no changes in distribution of spine phenotypes, as found by comparison of NT^−/−^ and NT^+/+^ mice.

Importantly, our data are in line with the hypothesis that agrin-22 signaling relies on its neuronal receptor α3NKA [10], as we observed an extensive overlap between α3NKA and “agrin puncta” expression in the *stratum radiatum* of CA1. In addition, we confirmed that AAV-Ag22 was properly delivered and expressed at synaptic sites, as both VGLUT1 and PSD95 colocalized with Ag22-scarlet at synapses. Moreover, we found that EGFP-expressing presynaptic boutons were larger and more numerous after Ag22 treatment and VGLUT1-positive puncta colocalizing with Ag22-scarlet were significantly larger than those without Ag22-scarlet colocalization. This observation suggests that AAV-Ag22 may also induce presynaptic growth in addition to postsynaptic changes. Agrin receptor α3NKA has been suggested to be localized both, pre- and postsynaptically [10]. Upon agrin binding, α3NKA activity is inhibited, favoring membrane depolarization and triggering Ca^2+^ influx through voltage-gated Ca^2+^ channels [39]. Subsequent activation of Ca^2+^ effectors like CaMKII may trigger a sequence of events that lead to the generation of new actin filaments and, thus, morphological changes of synapses and the formation of filopodia [39, 62]. Overall, these results provide in vivo evidence of the synaptogenic effects of agrin-22, the product of NT-dependent agrin cleavage, and suggest that it might be an important mechanism for synaptic plasticity, and synapse formation and maturation.

## Methods

### Animals

All experiments and behavioral procedures were conducted in accordance with animal research ethics standards defined by German law and approved by the Ethical Committee on Animal Health and Care of the State of Saxony-Anhalt (TVA 2502–2-1159 and 42,502–2-1343).

Mice constitutively lacking exons 10 and 11 from the NT gene (NT^−/−^) (Reif et al., 2007) and their wild-type littermates (NT^+/+^) were backcrossed to C57BL/6 J mice for > 9 generations. NT^−/−^ and NT^+/+^ mice for experiments were obtained by mating male and female NT^+/−^ mice. NT mice were kindly provided by Dr. Peter Sonderegger from the University of Zurich. Heterozygous neurotrypsin (NT^+/−^) mice were crossbred with Thy1-EGFP-M^+/−^ mice, which were purchased from Jackson Laboratory (www.jax.org/strain/007788). The NT^+/+^/Thy1-EGFP-M^+/^* and NT^−/−^/Thy1-EGFP-M^+/^* mice (+ /_*_ stands for + / + or +/−) used for spine analysis were obtained by mating male and female NT^+/−^/Thy1-EGFP-M^+/−^ mice.

C57BL/6 J, NT, Thy1-EGFP-M, and NT/Thy1-EGFP-M mice were bred at the animal facility of DZNE Magdeburg. For electrophysiological experiments, we used 4-week-old NT^−/−^ and NT^+/+^ mice of both sexes. For behavioral experiments, we used “juvenile” (3- to 5-week-old) male littermates or a cohort of aged (11- to 24-month-old age-matched; on average, 18 months old) NT^−/−^ and NT^+/+^ male mice. For immunohistochemistry and spine imaging, we used 3- to 4-week-old NT^−/−^/Thy1-EGFP-M^+/^* and NT^+/+^/Thy1-EGFP-M^+/^* mice of both sexes [for spine analysis associated behavioral test (contextual fear conditioning test), data collected in male juvenile mice are presented in Fig. 2i]. For viral injections, we used NT^−/−^/Thy1-EGFP-M^+/^* P7 mice of both sexes.

Mice were kept in a reversed light–dark cycle (12:12 h, light on at 9:00 pm) with access to food and water ad libitum and were transferred to fresh cages weekly. All behavioral experiments were carried out during the dark phase of the cycle, i.e., when mice are active.

For behavioral and spine imaging experiments, mice were individually housed 7 days before the start of the experiments. For electrophysiological experiments, mice were housed in groups of three-to-four mice per home cage. Behavioral analysis in matured mice was performed by an experimenter blinded to group identity. After the open field test, a few juvenile mice of both genotypes were excluded from further cognitive behavioral tasks, as they were not properly habituated to the arena and showed signs of nervousness, anxiety and agitation, most likely due to their young age. The numbers of mice used for each experiment are given in the figure legends. Outliers were excluded from graphs and subsequent statistical analysis using GraphPad outlier calculator software (www.graphpad.com/quickcalcs/Grubbs1.cfm). For spine imaging experiments, mice from the same litter were randomly allocated into three experimental groups (naïve, contextual fear-conditioned, and treated for extinction). For viral injections, littermates were randomly allocated into two experimental groups (AAV-Ag15 or AAV-Ag22). Injections were performed as follows: 1 mouse was injected with AAV-Ag15, and then, 1 mouse was injected with AAV-Ag22, etc. On postnatal day 7, mice were randomly picked up by their tail from the nest.

### Electrophysiological recordings in hippocampal slices

Acute hippocampal slices were prepared from 4-week-old NT^−/−^ and NT^+/+^ mice. Each mouse was killed by cervical dislocation, followed by decapitation. The brain was removed from the skull and transferred into ice-cold artificial cerebrospinal fluid (ACSF) saturated with carbogen (95% O_2_/5% CO_2_) containing (in mM) 250 sucrose, 25.6 NaHCO_3_, 10 glucose, 4.9 KCl, 1.25 KH_2_PO_4_, 2 CaCl_2_, and 2.0 MgSO_4_ (pH = 7.3). Both hippocampi were dissected out and sliced transversally (400 µm) using a tissue chopper with a cooled stage (custom-made by LIN, Magdeburg, Germany). Slices were kept at room temperature in carbogen-bubbled ACSF (95% O_2_ /5% CO_2_) containing 124 mM NaCl instead of 250 mM sucrose for at least 2 h before recordings were initiated.

Recordings were performed in the same solution in a submerged chamber that was continuously superfused with carbogen-bubbled ACSF (1.2 ml/min) at 32 °C. Recordings of field excitatory postsynaptic potentials (fEPSPs) were performed in CA1a and CA1c with a glass pipette filled with ACSF to activate synapses in the CA1b *stratum radiatum*. The resistance of the pipette was 1–4 MΩ. Stimulation pulses were applied to Schaffer collaterals via a monopolar, electrolytically sharpened and lacquer-coated stainless-steel electrode located approximately 300 mm closer to the CA3 subfield than to the recording electrode. Basal synaptic transmission was monitored at 0.05 Hz and collected at 3 pulses/min. The spaced LTP protocol was performed as previously described (Kramár et al., 2012). LTP was induced by applying 5xTBS with an interval of 20 s. One TBS consisted of a single train of ten bursts (four pulses at 100 Hz) separated by 200 ms and the width of the single pulses was 0.2 ms. To induce spaced LTP, we applied two trains of TBS (TBS1/TBS2) separated by 1 h. The stimulation strength was set to provide baseline fEPSPs with slopes of approximately 50% of the subthreshold maximum. The data were recorded at a sampling rate of 10 kHz and then filtered (0–5 kHz) and analyzed using IntraCell software (custom-made, LIN Magdeburg, Germany).

### Behavioral tests

All experiments were performed under uniform illumination (30 lx, unless otherwise stated), and all behavior was video recorded using a USB video camera and analyzed using ANY-maze software (ANY-maze, version 4.99, Stoelting Co., Wood Dale, IL). All recorded movies were analyzed by a trained observer blinded to the groups.

In juvenile male mice, the following behavioral tests were performed: open field test, novel object recognition test, three-chamber sociability test, and conventional and spaced contextual fear conditioning (using two different cohorts of mice). To characterize the persistence of behavioral changes found in juvenile mice and to further extend the behavioral characterization of NT^−/−^ mice, a battery of eight behavioral tests was performed using one cohort of aged male mice. The test battery included the open field test, novel object location test, novel object recognition test, temporal order recognition test, three-chamber sociability test, three-chamber social recognition test, elevated plus maze test, and contextual fear conditioning (CFC). The order of the tests was optimized according to the degree of invasiveness to reduce the chance that prior tests would influence animal performance in later tests [63]. Because the cohort of matured mice included mice of varying ages, covariance analysis with age as a covariate was performed for all behavioral tests. Because no effect of age was revealed (Suppl. Data 2), data for all ages were pooled and analyzed by ANOVA.

#### Open field test

The open field apparatus was made out of white polyacrylics and consisted of a white-square arena (50 × 50 × 30 cm). Experimental subjects were carried to the testing room in their home cages at least 30 min before the beginning of the experiment. Mice were placed in the center of the open field and allowed to freely explore it for 10 min. In juvenile mice, the first 5 min were used for the analysis of the following behavioral parameters: time spent in the inner area (central zone, 30 × 30 cm) and in the outer area (periphery) of the open field arena, locomotor activity (total travel distance), average speed, immobility time, grooming activity (including washing or mouthing of forelimbs, hind paws, face, body, and genitals), and number of defecations (number of fecal boli produced). The protocol for the open field test in matured mice was the same as that used for young mice. However, since the elevated plus maze test was performed to evaluate the anxiety status of the mice, parameters that highly depend on manual counting were not analyzed. Before the start of each session and between animals, the open field test apparatus was carefully wiped with a 70% alcohol solution.

#### New object location test

The test started 24 h after the open field test in matured mice. The same apparatus (50 × 50 × 30 cm) used for the open field test was used in the NOLT. Two pieces of A4 paper with stripe patterns were stuck to the upper middle area on two adjacent walls and served as landmarks. Experimental subjects were carried to the testing room in their home cages at least 30 min before the beginning of the experiment. The experiment included two phases: the encoding phase and the retrieval phase. During the encoding phase, two identical objects were placed at the adjacent corners of the central area (30 × 30 cm) of the open field arena; one of them was close to the corner which had landmarks on both walls. During the retrieval phase, this object was moved to the adjacent corner of the central part in the open field area to have both objects in a diagonal configuration. In both phases, mice were given 10 min for free exploration. In the same trial, objects were counterbalanced between mice. In different trials, different pairs of objects were used. The interval between the encoding and retrieval phases was 24 h. Before placing the next animal in the arena, the apparatus was carefully wiped with a 70% alcohol solution. The exploration time for each object was automatically counted by ANY-maze software. Exploration was considered to occur when the animal’s head was at a distance < 2 cm from the object but excluded time intervals when the animal climbed onto the object. The discrimination ratio was calculated as [time exploring the object in a novel position—time exploring the object in a familiar position]/[time exploring the object in a novel position + time exploring the object in a familiar position] × 100%.

#### Novel object recognition test

The same apparatus used for the open field test was used in the novel object recognition test. Experimental subjects were carried to the testing room in their home cages at least 30 min before the beginning of the experiment. The test was performed using a standard protocol [64] that included two phases: a familiarization/encoding (F) phase and a test/retrieval (R) phase. Juvenile mice were habituated to the apparatus 2 days before familiarization for 10 min each day. In matured mice, the test started 24 h after the novel location recognition test, which served as the encoding phase of the novel object recognition test. During familiarization, mice were placed in the arena for 10 min and were allowed to freely explore two identical objects separated by 25 cm in the center of the arena. During the retrieval phase, one familiar object and one novel object were placed in the center of the arena and mice were allowed to explore the apparatus for 10 min. In the same trial, objects were counterbalanced between mice; between trials, different sets of objects were used. The interval between the encoding and retrieval phases was 24 h. Before the start of each session and prior to bringing the next animal into the arena, the apparatus was carefully wiped with a 70% alcohol solution. The exploration time for each object and the total exploration time were manually estimated. Exploration was considered to have occurred when the orientation of the animal’s nose was at a distance < 2 cm from the object and included the time spent sniffing and directly touching the object. Novelty detection was evaluated by calculating the discrimination ratio as follows: [novel object time – familiar object time]/[novel object time + familiar object time] × 100%.

#### Temporal order recognition test

Matured mice were evaluated by the temporal order recognition test. The test was initiated 24 h after the novel object recognition test using the same test chamber and location of objects. This test included two encoding phases and a retrieval phase. In each encoding phase, a pair of novel identical objects were introduced to animals; in the retrieval phase, one object from each pair was used to test if animals could recognize the temporal order of objects. Objects and the relative position of objects did not change in the retrieval phase and were counterbalanced for animal genotype. The interval between any two consecutive test phases was 1.5 h. In all three phases, animals were given 10 min for free exploration. The same criteria as in NOLT that were used to define exploration time were applied for automatic analysis by ANY-maze software. The discrimination ratio was calculated as [exploration time of the object less recently shown – exploration time of the object recently shown]/[exploration time of the object less recently shown + exploration time of the object recently shown] × 100%.

#### Sociability test

Sociability levels were assessed using a three-chamber apparatus (60 × 30 × 30 cm) made out of white polyacrylics and that had connecting doors between chambers. The test in juvenile mice was performed using a standard protocol [65]: mice were habituated to the apparatus 2 h before the test for 10 min. Subsequently, one “stimulus” mouse was placed inside a small cage at one end of the compartments and an identical empty cage was placed in the opposite compartment. The mouse performing the test was allowed to explore the whole apparatus for 10 min. The exploration times spent near the cage containing the stimulus mouse and near the empty cage were estimated. The time spent sniffing and directly touching the mouse and the empty cage was considered as exploration time. In juvenile mice, as active social interaction is difficult to track automatically and hard to distinguish from time spent merely in proximity to the social partner, social interaction was analyzed and scored manually by an experimenter who was blind to the mouse genotype. In the three-chamber sociability test, the discrimination ratio was calculated as follows: [stimulus mouse time – empty cage time]/[stimulus mouse time + empty cage time] × 100%. Before the start of each session and before placing the next animal into the apparatus, the three-chamber sociability test apparatus was carefully wiped with a 70% alcohol solution; stimulus mice were changed every two sessions to avoid anxiety and stress.

In matured mice, the sociability test was initiated 24 h after the temporal order recognition test. The protocol was similar to that used for young mice. A few settings were optimized to improve the quality and reliability of automatic analysis: 1) the identical small cages that would be used as a potential container of “stimulus animals'' were set on both terminal compartments in the phase when an animal was allowed to familiarize itself with the whole test environment (to reduce the exploration triggered by small cages); 2) during the test phase, a novel object was put into the small cage which had no animal inside as a stimulus (to better control the preference of subject mice for alive mice than for a nonanimated object); 3) a novel stimulus mouse was introduced after every four consecutive trials (to maintain the mental status of stimulus mice); and 4) the small cages were covered from above (to reduce the chance that the ANY-maze software might mistakenly track stimulus mouse movement rather than subject mouse movement, leading to the creation of an artifact). After these optimizations, the automatic analysis was precise enough to track the movements of subject mice and reflect their interests in other animals. Exploration was considered to have occurred when the orientation of the animal’s head was at a distance < 2 cm from the cages but excluded instances when the animal climbed onto the small cages.

#### Social recognition test

The test was performed in matured mice in the three-chamber maze and was initiated 4 h after the sociability test using similar basic settings. The familiar stimulus mouse was placed inside the same small cage at the same end of the compartments as in the sociability test, and a novel stimulus mouse was placed inside the opposite compartment. Mice performing the test were given 10 min to freely explore the three-chamber apparatus. Before placing the next subject animal in the apparatus, the apparatus was carefully wiped with a 70% alcohol solution. Stimulus animals were changed every four consecutive trials. The exploration time toward cages including novel or familiar stimulus animals was automatically measured by ANY-maze software using the same criteria as was used in the sociability test. The discrimination ratio was calculated as [time exploring the cage containing the novel stimulus animal—time exploring the cage containing the familiar stimulus animal]/[time exploring the cage containing the novel stimulus animal + time exploring the cage containing the familiar stimulus animal] × 100%.

#### Elevated plus maze test (EPM)

The EPM was performed on the day after SRT in matured mice. The maze consisted of four arms in the shape of a cross (length: 30 cm, width: 5 cm, and height from floor: 50 cm). Two arms were enclosed by 15.25 cm-high walls and faced each other on opposite sides; the other two arms were open borders [66]. To reduce behavioral bias introduced by the light–dark difference between open and enclosed arms, LED lamps were used to guarantee that the light was reflected from the ceiling over enclosed arms. The final light intensity readouts at the end of all four arms were 15 lx. The movement of mice was evaluated in a single 10-min session. The heads of the mice were tracked by ANY-maze software, so that entry into open arms and enclosed arms could be noted. The time animals’ heads were located in different arms as well as the discrimination ratio [(time in enclosed arms—time in open arms)/(time in closed arms + time in open arms) × 100%] were used to evaluate anxiety status in mice.

#### Spaced contextual fear conditioning (CFC)

A *spaced* contextual fear conditioning (CFC) paradigm was performed as previously described [67] but with two conditioning sessions separated by 1 h instead of having only one session. Before CFC training, mice were handled and habituated to the experimental room, and the conditions were maintained in a home cage for 3 days for 5 min each day. During the training day (day 0, d0), CFC was performed as follows: mice were placed into a neutral context (CC-) (the freezing level during this time interval was taken as the baseline value for the CC-) for 5 min; 2 h later, mice were placed into a conditioned context (CC +) and 3 × medium intensity footshocks were applied (0.5 mA, 1 s) with an interval of 30 s. This procedure was repeated 1 h later (hence, spaced learning was employed). The protocol included 1 min exploration in the CC + before the first shock was administered (the freezing level during this time interval was taken as the baseline value for the CC +), 30 s after the second shock, and again 30 s after the third shock. Mice were then left for additional 30 s in the CC + before being transferred to a home cage. The CC + was a chamber (20 × 20 × 30 cm) with a contrast black-and-white chess-like pattern on the walls and a metal grid on the floor. The neutral context (CC −) was the same chamber, but with gray walls and a gray plastic floor. Before the start of each session and before placing the next animal in the apparatus, the fear conditioning apparatus was carefully wiped with a 75% alcohol solution (CC +) or with Meliseptol® having a different smell (CC-) to facilitate discrimination between both contexts. In juvenile mice, memory retrieval was tested at d2, and mice were placed in the CC + for 5 min to assess the retention of contextual memory. Subsequently, 9 × memory extinction sessions were performed on 3 consecutive days (d5-d7, 3 × sessions per day). In each session, mice were placed in the CC + for 5 min. At d9, mice were placed in the CC + again for 5 min (second memory retrieval), and freezing was assessed to evaluate fear memory extinction. A computerized fear conditioning system (Ugo Basile, Gemonio, Italy) was used for analysis. The total freezing time was manually calculated as the percentage of 5 min (in either of the two contexts, CC + and CC-) when animals showed no movement except for breathing. The discrimination ratio was calculated as follows: [freezing time in the CC + – freezing time in CC-]/[freezing time in the CC +  + freezing time in CC-] × 100%.

#### Contextual fear conditioning

Classical (non-spaced) CFC was performed as above but with the following modifications. In matured mice, CFC was performed on the day after EPM. During the training day (d0), mice experienced the CC- and CC + for 5 min each, only once. The interval between the CC- and CC + was 2 h. The 0.6 mA footshock with a duration of 1 s was administered in the CC + at 120, 180, and 240 s. During the first recall day (d1), mice were placed in the CC + and CC- for 5 min to confirm the contextual fear memory. From day 2 to day 4, 9 × memory extinction sessions were performed (3 × sessions per day) by placing mice in the CC + for 5 min. On the second recall day (d5), mice were placed in the CC + and CC- for 5 min to confirm the extinction of contextual fear memory. For juvenile mice, the intensity of foot shocks were slightly lower than that administered to mature mice (0.5 mA), and no CC- was present.

### Spine analysis

#### Sample collection, perfusion, and tissue processing in juvenile mice

Juvenile mice were individually anesthetized with 100 mg/kg ketamine and 5 mg/kg xylazine and transcardially perfused with 0.1 M phosphate buffer solution (PBS, pH 7.4) followed by 4% formaldehyde diluted in 0.1 M phosphate buffer solution for 15 min. Brains were removed and postfixed for 24 h in 4% formaldehyde-PBS at 4 °C. The brains were then transferred to a sucrose solution (1 M in 0.1 M NaH_2_PO_4_ buffer) until the solution had infiltrated into the whole brain (~ 48 h) to cryoprotect the tissue. Finally, the brains were frozen in 100% 2-methylbutane at -80 °C and cryosectioned in 50-μm-thick coronal sections. Floating sections were kept in cryoprotective solution (1 part ethyl glycol, 1 part glycerin, and 2 parts PBS, pH 7.4). All sections were washed 3 × in 0.1 M phosphate buffer solution (PBS, pH 7.4) for 10 min with gentle shaking. Subsequently, sections were briefly washed in ddH_2_O to remove salts from PBS and mounted on SuperFrost glasses with Fluoromount (Sigma F4680).

#### Golgi–Cox staining in aged mice

Tissue preparation started 24 h after the end of extinction test in matured mice. For each genotype, 6 mice were used. Mice were anesthetized with 3% isoflurane and decapitated. Brains were quickly removed from the skull and washed with ddH_2_O to remove blood from the surface. Golgi–Cox impregnation of neurons was performed using the FD Rapid GolgiStain™ kit (FD NeuroTechnologies, # PK401)[68]. After a 3-week incubation, dye-impregnated brains were rapidly frozen in isopentane at -50 °C and then stored at -80 °C. For cryosection, brains were embedded in TissueTek O.C.T. compound (Sakura Finetek, # 4583) and coronally cryosectioned in 100-µm thickness and directly mounted on gelatin-coated slides (FD NeuroTechnologies, # PO101) with the help of solution C provided in the kit. Sections were stained according to the manufacturer’s protocol and mounted using the ROTI®Histokitt embedding medium (Carl Roth, # 6638)[68].

#### Spine imaging and deconvolution

For imaging, dendrites were selected from slices containing the dorsal hippocampal area. Images were acquired by an experimenter blinded to genotype information using a confocal laser-scanning microscope (LSM 700 and LSM 780 for samples from juvenile and aged brains, respectively; Carl Zeiss, Germany) and Zen software (Carl Zeiss, Jena, Germany). Secondary apical dendrites from CA1 pyramidal neurons were imaged for spine analysis.

For analysis of spines in juvenile brains, we used NT/Thy1-EGFP-M mice that express EGFP in a fraction of pyramidal cells. Z-stacks were collected with 0.21 µm interval using a 488 nm laser and a 63 × oil objective (NA = 1.4) with a 2.6 × optical zoom. The voxel size was 0.0644 × 0.0644 × 0.2065 µm. Deconvolution of images was performed using Huygens deconvolution software (Scientific Volume Imaging). The images were deconvolved using the “Classic Maximum Likelihood Estimation (CMLE)” algorithm implemented in Huygens software (Scientific Volume Imaging), set with 50 iterations, a quality threshold of 0.01, and a signal-to-noise ratio value of 25. A theoretical point spread function was used.

For analysis of spines in aged brains, Z-stacks were collected with 0.25 µm interval using a 405 nm laser, a 63 × oil objective (NA = 1.4), and 2 × digitally zoomed. The final voxel size was 0.066 × 0.066 × 0.250 μm. Images were rotated and cropped in ImageJ software to focus on target secondary apical dendrites and also set the root of each dendrite at the right edge of image for further analysis. To facilitate visual inspection of the Z-projections in Fig. 6A, the background was removed using the rolling ball method (radius = 30 px) and images were corrected using an unsharp mask filter (radius = 2 px, mask weight = 0.7) in Fiji software.

#### Spine density and morphology analysis

To identify, classify, and count GFP-labeled dendritic spines in juvenile mice, images were morphometrically analyzed using NeuronStudio software (CNIC, Mount Sinai School of Medicine, New York, NY, USA) and a custom-written Excel worksheet template to analyze the parameters provided by the NeuronStudio software. The analysis was performed by an experimenter blinded to group identity. Measurements started with some interval after the branching point after which the spine density appeared as stable. Spines along the dendrites were assessed using standard parameters for the distinction of stubby-, filopodia-like/thin-, and mushroom-type spines, as previously described [69, 70]. Parameters were set as suggested [69]. Only protrusions with a clear connection of the head of the spine to the shaft of the dendrite were counted as spines. In addition, a visual examination was also used to detect false ‘‘spine calls’’. This systematic approach was chosen to account for possible changes in spine distribution along dendrites.

Morphometric analysis of Golgi–Cox labeled dendritic spines in aged mice was performed as described previously [71–75]. Briefly, the images were semiautomatically analyzed using SpineMagick software [71] (the code is available in https://doi.org/10.5281/zenodo.6114928). To perform morphological analysis of images, we inverted the image intensity scale, and enhanced the contrast to 0.1% saturated pixels using ImageJ software. Next, spatial Gaussian blurring with Sigma(radius) = 1 was applied to decrease the level of noise. Subsequently, we performed image projections onto z plane, cropping the dendrite segments in the z-direction, to reduce the amount of projected artifacts from the background. For some images, it was possible to crop the entire dendrite by the single cube; in other cases, the dendrite segment was not straight in the z-direction and it was necessary to use few cubes to crop it properly. The spine density was calculated by dividing the number of spines by the length of the marked dendritic segment. Dendritic spines were counted manually by scrolling through the z-stacks of 3D images. That dendrite length value was interpolated using the SpineMagick.

Length (L), head width (H), and neck width (N) measured from SpineMagick were adopted for dendritic spine classification [76–78]. Spines were classified into mushroom spines (H/N > 1.3); stubby spines (H/N ≤ 1.3, and L/N ≤ 1.1); branched spines were excluded from quantitative morphological analysis; the rest spines were recognized as thin spines. The cutoff values were decided based on previous publications when genotype information was blinded [76–78].

### Generation of agrin-expression vectors and adeno-associated viral (AAV) particles

Full-length (GeneID: 11,603) agrin constructs were obtained from Dharmacon (accession: BC150703). The DNA sequence corresponding to the 22 kDa C-terminus of agrin was used to induce spinogenesis and filopodia as previously described [7], while the 15 kDa C-terminus sequence was used as a control [10]. The cDNA was amplified using primers (sequences of the primers can be found in supplementary data, Table S2) and cloned into an AAV vector where the gene was expressed under the synapsin promoter and fused at the N-terminus of the red fluorescent reporter protein scarlet [79]. To secrete agrin fragments into the extracellular environment, we additionally cloned a secretion signal sequence from the receptor protein tyrosine phosphatase sigma at the N-terminus of the agrin sequence as described previously [80].

AAV particles were produced as previously described [81] with minor modifications. Briefly, HEK 293 T cells were transfected using the calcium phosphate method with an equimolar mixture of the expression plasmid, pHelper plasmid and RapCap plasmid DJ. After 48 h of transfection, cells were lysed using freeze–thaw cycles and treated with benzonase at a final concentration of 50 units/ml for 1 h at 37 °C. The lysate was centrifuged at 8000 g at 4 °C. The supernatant was collected and filtered with a 0.2-micron filter. The filtered supernatant was passed through pre-equilibrated Hitrap Heparin columns (Cat no. 17–0406-01; Ge HealthCare Life Science), followed by a wash with wash Buffer 1 (20 mM Tris, 100 mM NaCl, pH 8.0; filtered sterile). Columns were additionally washed with wash Buffer 2 (20 mM Tris 250 mM NaCl, pH 8.0; filtered sterile). Viral particles were eluted with elution buffer (20 mM Tris 500 mM NaCl, pH 8.0; filtered sterile). Amicon Ultra-4 centrifugal filters (100 kD cutoff) were used to exchange the elution buffer with sterile PBS. Finally, viral particles were filtered through a 0.22 μm syringe filter (Sigma-Aldrich, product no. Z741696-100EA), aliquoted, and stored at -80 °C until required.

### AAV intrahippocampal injections

NT^−/−^/Thy1-EGFP-M^±^ mice of both sexes were anesthetized at postnatal day P7 with 3% isoflurane (Baxter, Germany) delivered as a mixture with O_2_ through a vaporizer (Matrx VIP 3000, Midmark, Versailles, USA) and a custom-made mouse breathing mask that was a suitable size for P7 mice. The cranial skin was locally disinfected and incised, the skull was exposed by displacement of the skin and muscles, and a small hole was drilled into the skull at the injection site. Craniotomy was performed on both hemispheres using stereotaxic information for external landmarks on the skull, such as lambda and bregma and to other distinct landmarks, such as characteristic blood vessels of the bone and the brain [82], which had to be adapted to the smaller size of the young skull and brain. The following coordinates were used to target the CA1 area: ML, 1 mm and DV, 1.2 mm. A total of 500 nl of viral suspension (1.84 × 10^11^ particles/ml) was injected per hemisphere using a pulled glass micropipette (World Precision Instruments, WPI, glass capillaries with product no. 4878) and a nanoliter injector (WPI, Nanoliter 2010). To prevent backflow, the micropipette was left in the brain for 5 min before it was pulled out. The scalp was closed and sutured, and then, the animals were allowed to recover on a heated pad. P7 pups were separated from the mother for a maximum of 3 h to prevent them from being rejected.

### Immunohistochemistry

Sample collection, animal perfusion, and tissue processing were performed as described above for spine analysis. For α3NKA, VGLUT1, and PSD95 immunolabeling, 40 μm free-floating sections were washed in PBS [3 × 10 min, at room temperature (RT) with gentle shaking] and incubated for 1 h (at RT with gentle shaking) in a blocking and permeabilizing solution containing 5% normal goat serum (NGS, Gibco, 16,210–064), 0.5% Triton X-100 (Sigma-Aldrich, T9284), and 0.1% Tween-20 (Roth, 9127.1) in PBS. Subsequently, slices were treated for 24 h (at 4 °C with gentle shaking) with the primary antibody in PBS containing 5% NGS, 0.5% Triton X-100, and 0.1% Tween-20. Anti-sodium potassium ATPase alpha 3 (mouse, dilution 1:250, XVIF9-G10, Novus Biologicals), anti-VGLUT1 (guinea pig, dilution 1:1000, 135,304, Synaptic Systems), and anti-PSD95 (mouse, dilution 1:500, Ab2723, Abcam) were used as primary antibodies. The slices were then washed 3 × for 10 min at RT in PBS containing 0.1% Triton X-100 and 0.1% Tween-20 (washing buffer) and incubated on a shaker for 3 h at RT with the secondary antibody. Secondary antibodies conjugated with Alexa 405 and 488 (Life Technologies) against the respective primary antibody were used with a dilution of 1:800 for Alexa 488 and 1:500 for Alexa 405. Afterward, slices were washed 3 × 10 min at RT with washing buffer and 1 × 10 min at RT with PBS, and then mounted on SuperFrost glass with Fluoromount (Sigma F4680).

### Image capturing and analysis of immunohistochemical data and presynaptic boutons

To analyze the size of VGLUT1-positive puncta colocalizing or not with Ag22-Scarlet, three independent images were selected for counting. Images were acquired using a confocal laser-scanning microscope (LSM 700, Carl Zeiss, Germany) and Zen software (Carl Zeiss, Jena, Germany). ImageJ 1.46 software (NIH, USA) was used for image analysis. For each image, channels were separated (Image > Color > Split channels). Then, thresholds were manually adjusted for each channel (Image > Adjust > Threshold (using Yen and Over/Under functions)). Subsequently, binary maps were created (Process > Binary > Make binary), and VGLUT1 puncta were recognized automatically as particles greater than 0.02 μm^2^ in the VGLUT1 channel (Analyze > Analyze particles > 0.02-Infinity). The size of each ROI was measured, and ROIs were superimposed on the binary map of the Ag22-Scarlet channel. ROIs were divided into two populations: ROIs with or without colocalization with AAV-Ag22 particles.

To analyze axonal bouton density and size after AAV-Ag15/22 injections in Thy1-GFP mice, Z-stack images were acquired in dorsal hippocampal CA1 area using SP8 microscope (Leica, Germany) using a 488 nm laser and a 63× oil objective (NA = 1.4). The voxel size was 0.045 × 0.045 × 0.25 µm. ImageJ was used for image processing and analysis. For each image, about three axons with clear bouton structures were selected, and then, images were maximumly projected and cropped for each axon. After the Gaussian blur filter was applied (sigma: 1.0), axons were traced with the neuroanatomy module of ImageJ (Plugins > Neuroanatomy > SNT). Bouton width was automatically masked with ImageJ [Analyze > Local Thickness > Local Thickness (masked, calibrated, silent)]. Based on this mask, binary maps were created for *en passant* boutons more than 0.45 μm (Process > Binary > Make binary); the boutons were further masked automatically as particles greater than 0.2 μm^2^ (Analyze > Analyze particles > 0.2-Infinity). The size of each ROI was measured, and ROIs were superimposed on the “Local Thickness” mask to read bouton width data. The bouton density of each axon was computed as ROI counts per 10 μm axonal length.

### Statistics

Statistical analysis of the results from behavioral tests in juvenile and matured mice was performed with SigmaPlot 13.0. A normality test (Shapiro–Wilk method) and an equal variance test (Brown–Forsythe method) were applied to determine which parametric test should be used. Grubbs' test (the extreme studentized deviate method) was applied to determine whether one of the values in the list is a significant outlier from the rest. For data obtained from repeated measures, a two-way RM ANOVA with the Holm–Šidák post hoc test was applied (CFC, LTP). For data not repeatedly acquired from many groups, two-way ANOVA with the Holm–Šidák post hoc test was applied (LTP, CFC associated spine analysis). For data collected from a single test based on novelty recognition (NOLT, NORT, TORT, Sociability, SRT), a two-sided paired t test was applied for analysis of exploring time. For other comparisons between two groups (discrimination ratios, datasets in OF, EPM, spine analysis in rescue experiment), a two-sided unpaired t test was applied. For comparison of datasets failed in the equal variance test (Figs. 6C, D, 7D, 8F (upper), Fig. 8G (upper), Fig. 8H (upper), Fig.S2B, and Fig.S4A), Welch's t test was applied. *P* < 0.05 was used to reject the null hypothesis. For comparison of cumulative distributions of dendritic spine parameters and axonal bouton analysis, the Kolmogorov–Smirnov (KS) test was used.

## Ethics approval

## Supplementary Information

Below is the link to the electronic supplementary material.

Supplementary file1 (XLSX 594 kb)

Supplementary file2 (XLSX 122 kb)

Supplementary file3 (DOCX 4178 kb)

## Data Availability

The source data and statistical analyses underlying all figures are provided as Source Data files. The datasets generated during and/or analyzed during the current study are available from the corresponding author upon reasonable request.
