# Supplementary material for: Mice deficient in synaptic protease neurotrypsin show impaired spaced long-term potentiation and blunted learning-induced modulation of dendritic spines
Source: Cell Mol Life Sci. 2023 Mar 5;80(4):82. doi: 10.1007/s00018-023-04720-z (PMC9986217; doi:10.1007/s00018-023-04720-z)
Supplement: Supplementary file 3 — Supplementary file3 (DOCX 4178 kb) [file 18_2023_4720_MOESM3_ESM.docx]

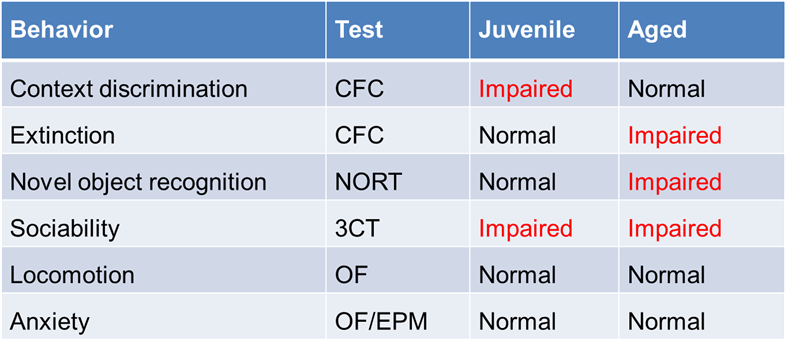


**Supplemental Table S1. Age-dependent changes in behavior of NT^-/-^ mice.** CFC, contextual fear conditioning; NORT, novel object recognition task; 3CT, three chamber task; OF, open field; EPM, elevated plus-maze.

| **Name** | **Sequence** |
| --- | --- |
| AAV_Syn_Agrin-22_Scarlet_WPRE_EcoR1_Fw | 5’-TAAGCAGAATTCGCCACCATGTCAGTGGGGGACCTAGAAACAC-3’ |
| AAV_Syn_Agrin-22/Agrin-15_Scarlet_WPRE_Xho1_Rev | 5’-TAAGCACTCGAGGAGAGTGGGGCAGGGTCTTAG-3’ |
| AAV_Syn_Agrin-15_Scarlet_WPRE_EcoR1_Fw | 5’-TAAGCAGAATTCGCCACCATGTGGATTGGAAAGGTTGGAGAACG-3’ |
| AAV_Syn_Sec(CPTX)_Agrin-22_Scarlet_WPRE_Age1_Fw | 5’-TAAGCAACCGGTTCAGTGGGGGACCTAGAAACAC-3’ |
| AAV_Syn_Sec(CPTX)_Agrin-22 and Agrin-15_Scarlet_WPRE_Xba1_Rev | 5’-TAAGCATCTAGACATATGGTCGACGAGCTCG-3’ |
| AAV_Syn_Sec(CPTX)_Agrin-15_Scarlet_WPRE_Age1_Fw | 5’-TAAGCAACCGGTTGGATTGGAAAGGTTGGAGAACG-3’ |

**Supplemental Table S2. Primers used for amplification of agrin constructs**

**
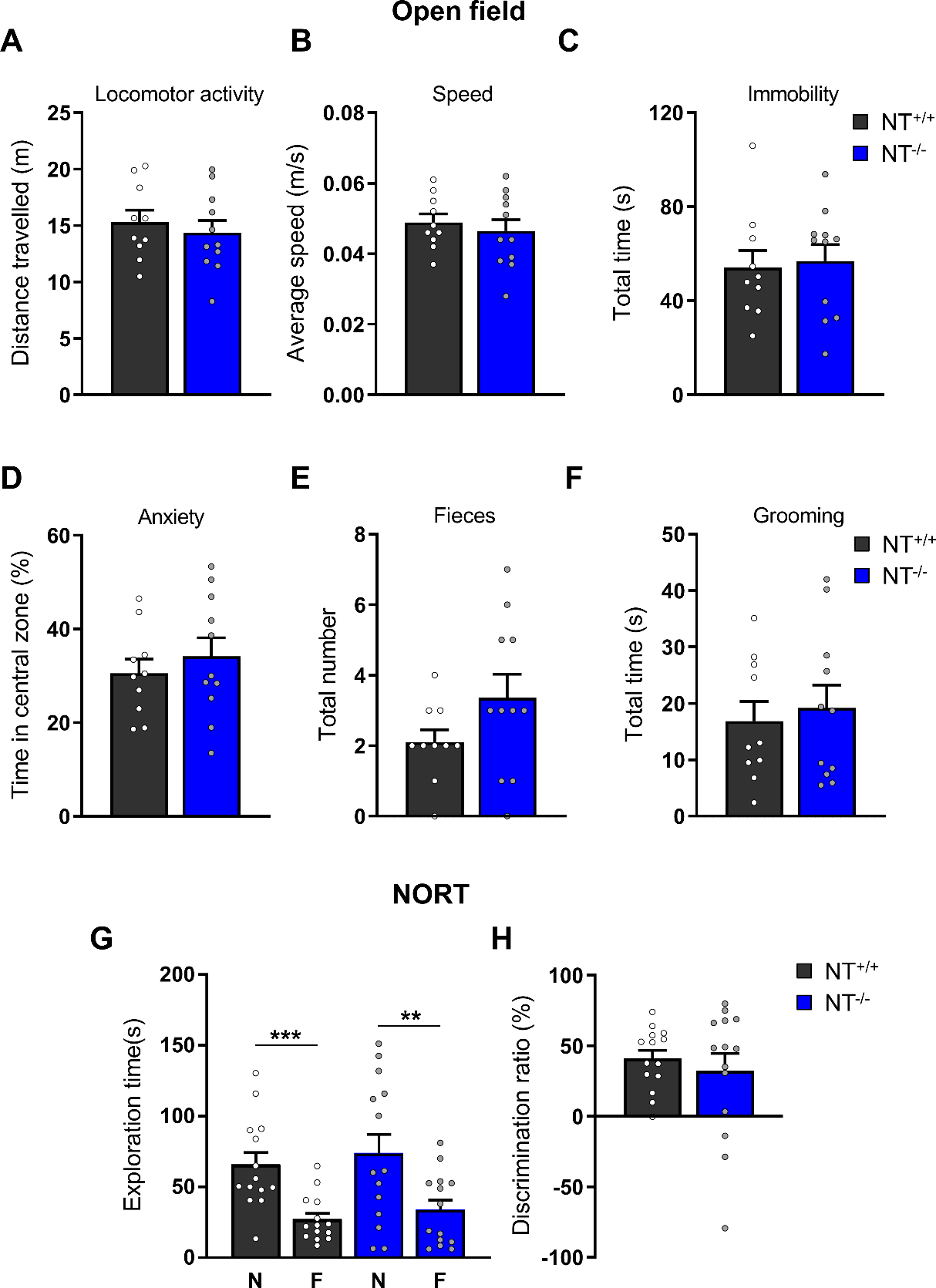
**

**Supplemental Figure S1. Behavioral parameters measured during the open field (OF) test and novel object recognition test (NORT) in juvenile NT^-/-^ and NT^+/+^ mice.** (A) The total distance traveled. (B) The average speed while traveling. (C) The total immobility time. (D)Time spent in the central zone of the arena. . (E) The number of fecal boli produced during the open field test. (F) The time spent grooming. . NT^+/+^: n = 10; NT^-/-^: n = 11. (G) Exploration time in the NORT. (H) Discrimination ratio in NORT. NT^+/+^: n = 14; NT^-/-^: n = 14. The data are presented as the mean ± SEM. ***P* < 0.01, ****P* < 0.001. Paired t-test was applied to compare exploration time within the same group for panel G; nonpaired t-test was used for other panels.

**
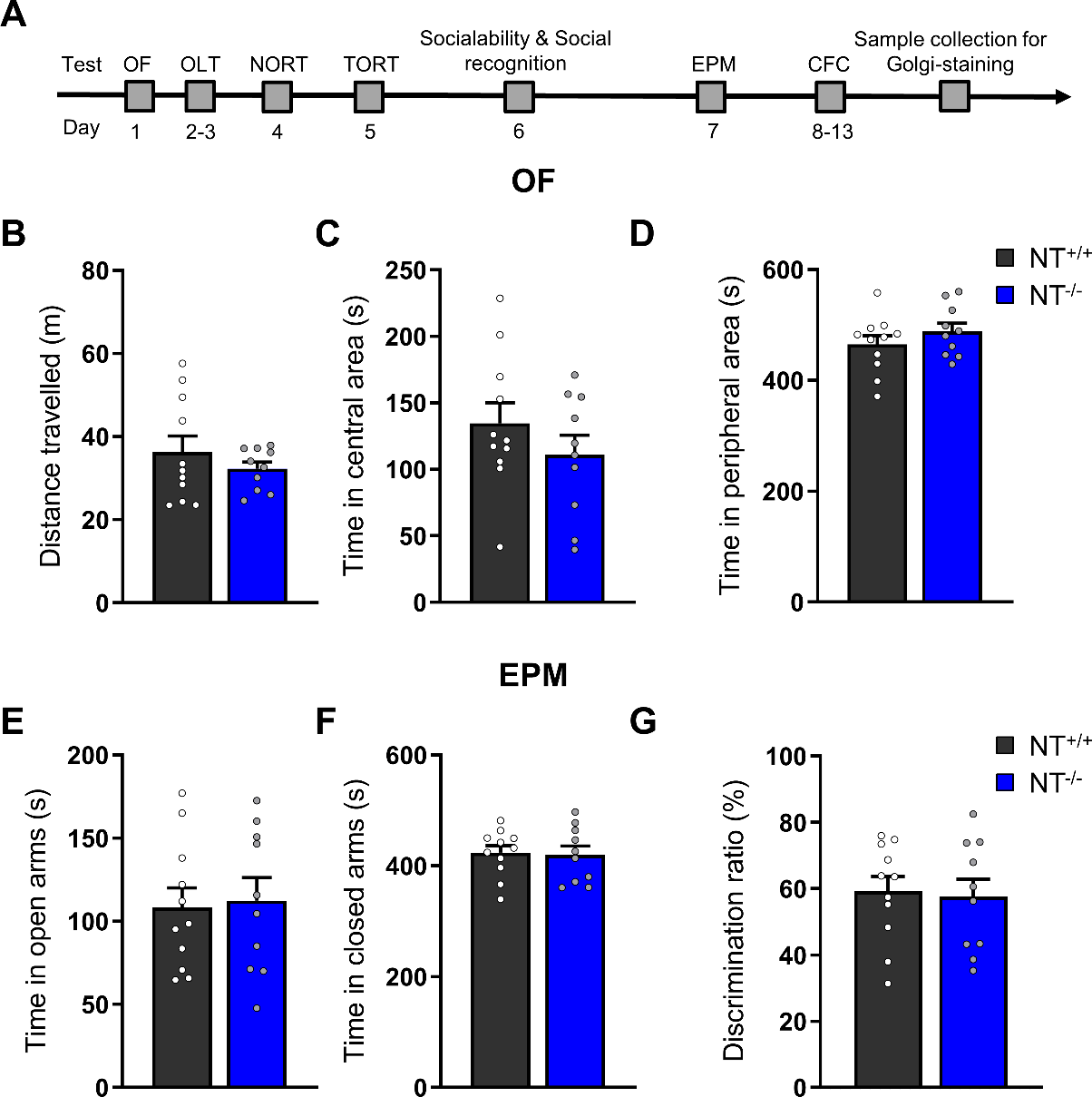
**

**Supplemental Figure S2. Behavioral parameters measured during the open field test and elevated cross maze test in aged NT^-/-^ and NT^+/+^ mice.** (A) The time course and the scheme of the experiment. (B) The total distance traveled in the open field. (C) Time spent in the central zone of the arena. (D) Time spent in the peripheral zone of the arena. (E) Time spent in the open arms of the elevated cross maze. (F) Time spent in the closed arms of the elevated cross maze. (G) Discrimination ratio calculated using time spent in arms. The data are shown as the mean ± SEM. NT^+/+^: n = 11; NT^-/-^: n = 10. Nonpaired t-test was used for comparison.

**
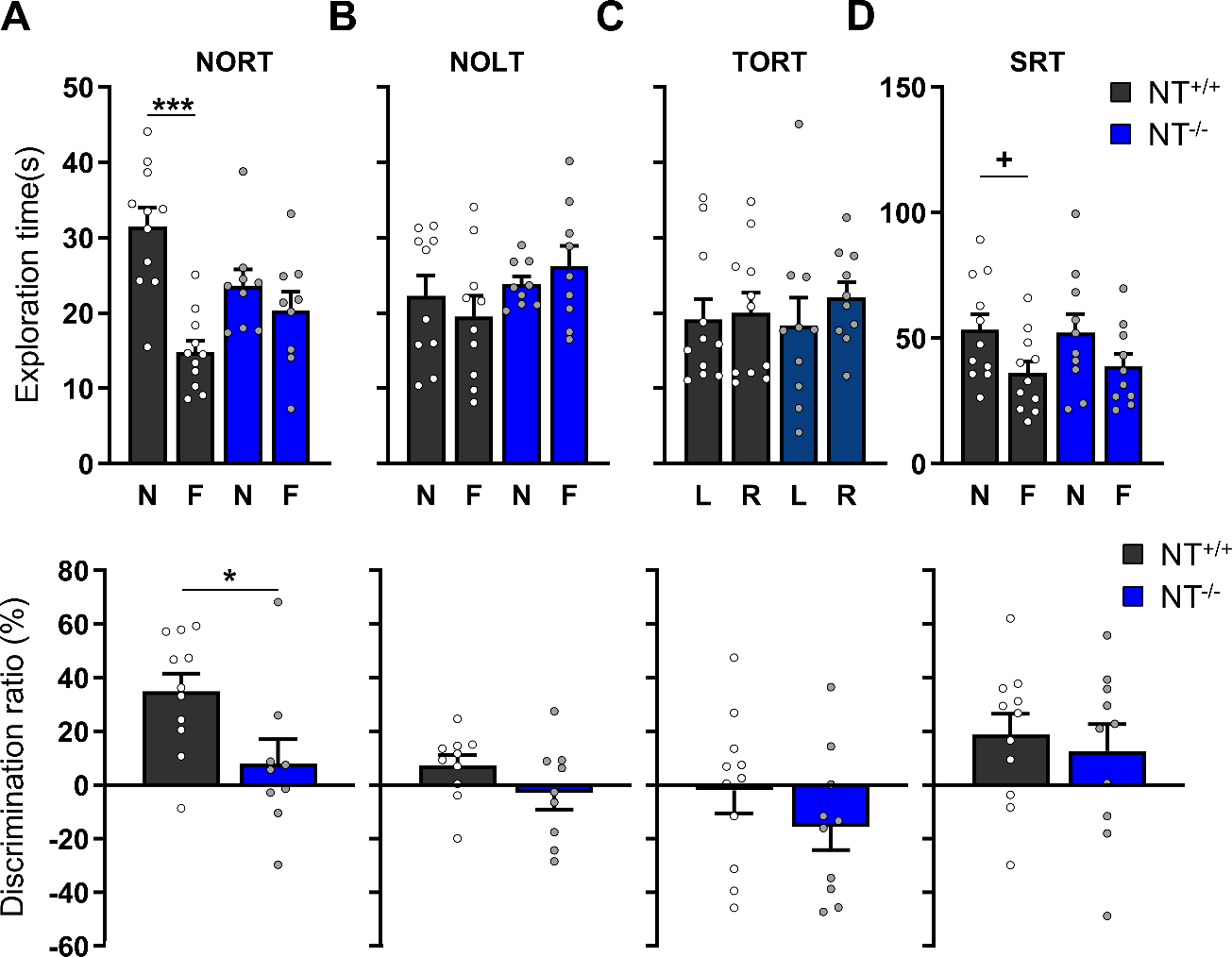
**

**Supplemental Figure S3. Aged NT^-/-^ and NT^+/+^ mice demonstrate impaired behavior in the novel object recognition test (NORT).** (A) Novel object recognition test (NORT), (B) novel object location test (NOLT), (C) temporal order recognition test (TORT) and (D) social recognition test (SRT); the corresponding discrimination ratios are shown in lower subpanels. **N**, novel object or animal; **F**, familiar object or animal; **L**, the object less recently shown; **R**, the object more recently shown. The data are shown as the mean ± SEM. NT^+/+^: n = 11; NT^-/-^: n = 10 (one animal was excluded in OLT and NORT based on the *a priori* set exclusion criteria: exploration for single object less than 5 s). ^+^*P < 0.1,* **P* < 0.05, ****P* < 0.001. Paired t-test was applied to compare exploration time within the same group for upper subpanels; nonpaired t-test was used for lower subpanels to compare the discrimination ratios between genotypes.

**
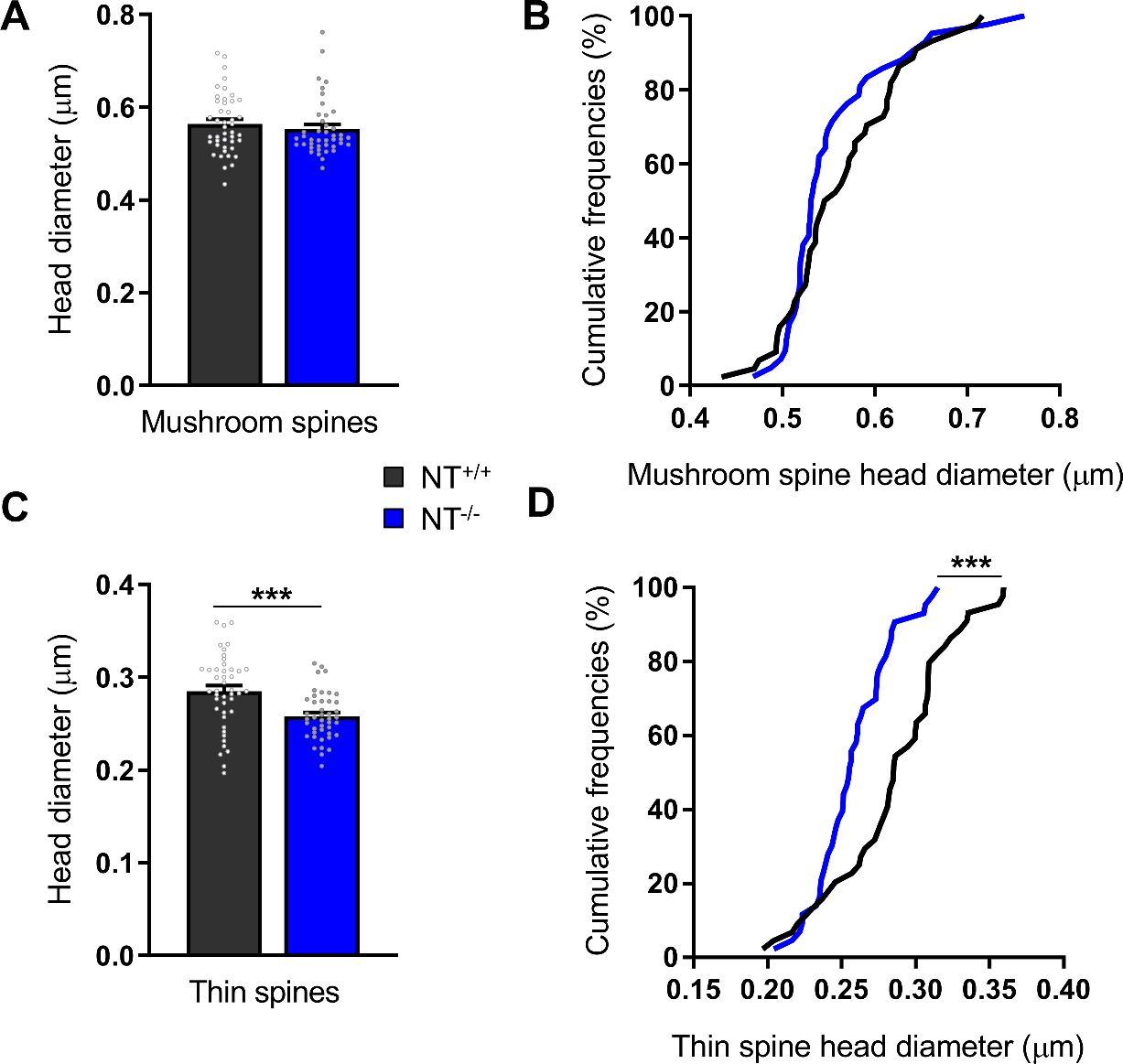
**

**Supplemental Figure S4. Juvenile NT^-/-^ mice have smaller diameter of thin spine heads after CFC compare to their NT^+/+^ littermates.** (A) The head diameter of mushroom spines was similar in both genotypes. (B) Cumulative frequency plot of the mushroom spine head diameter. (C) The head diameter of thin spines was significantly reduced in NT^-/-^ mice. (D) Cumulative frequency plot of the thin spines head diameter. To stress the difference between groups, the X-axes in C and D begin at a value greater than 0. The data are shown as the mean ± SEM. The numbers of analyzed NT^+/+^ and NT^-/-^ dendrites/mice were 44/5 (NT^+/+^ CFC) and 42/5 (NT^-/-^ CFC), respectively. ****P* < 0.001; statistical methods applied: Mann-Whitney rank sum test (A), nonpaired t-test (B) and Kolmogorov-Smirnov test (C, D).

**
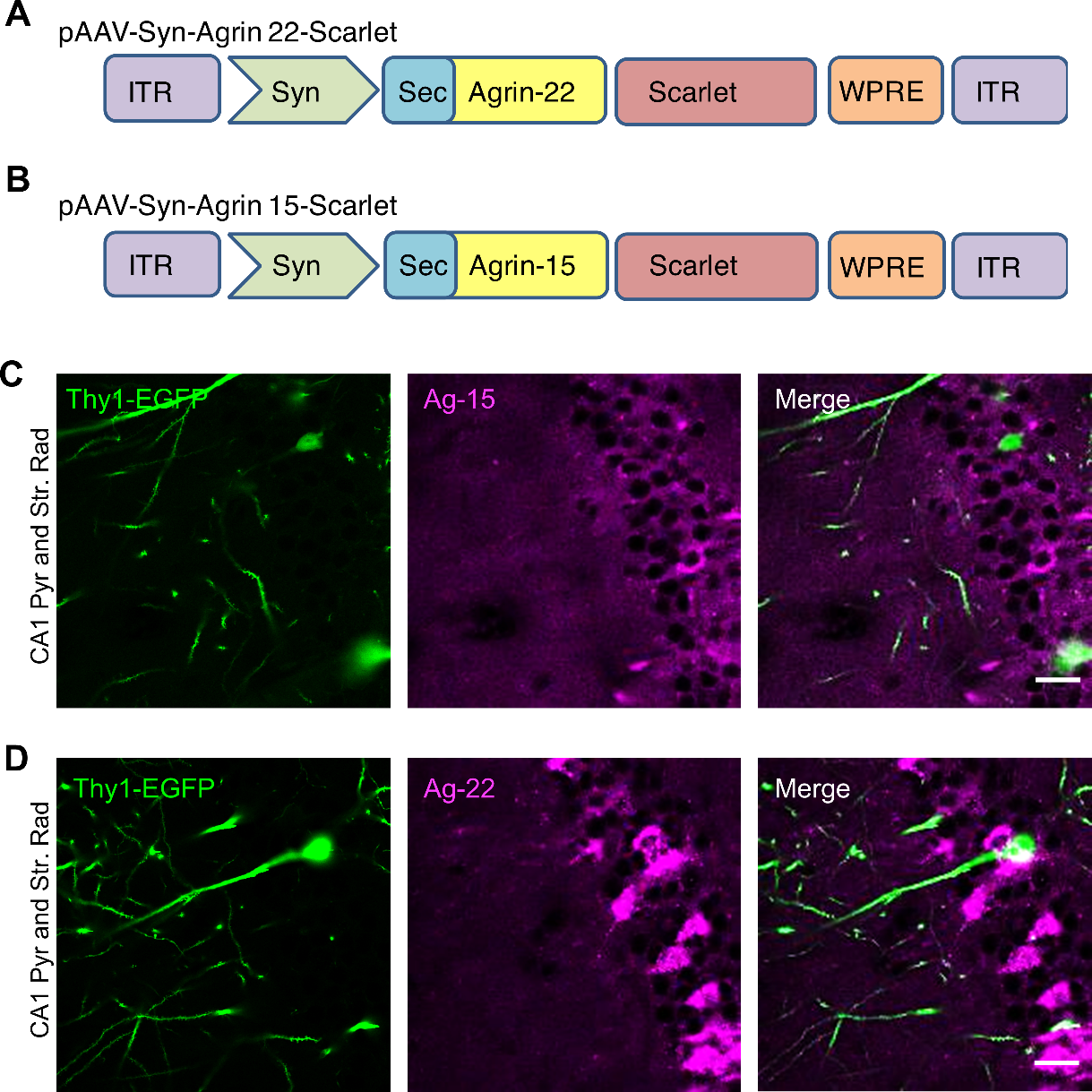
**

**Supplemental Figure S5. Schematic of the two AAV vectors used in this study and representative sections showing the expression of Ag22-scarlet and Ag15-scarlet in juvenile mice.** (A) AAV-Ag22 and (B) AAV-Ag15. The cassette contains agrin-22 or agrin-15 driven by the synapsin I (Syn) promoter, a secretion signal sequence (Sec) and the reporter red fluorescent protein scarlet. (C) Representative hippocampal section showing Ag15-scarlet expression in the CA1 *stratum pyramidale* and the *stratum radiatum*. Scale bar, 20 μm. (D) A representative hippocampal section showing Ag22-scarlet expression in the CA1 *stratum pyramidale* and the *stratum radiatum*. Scale bar, 30 μm.

**
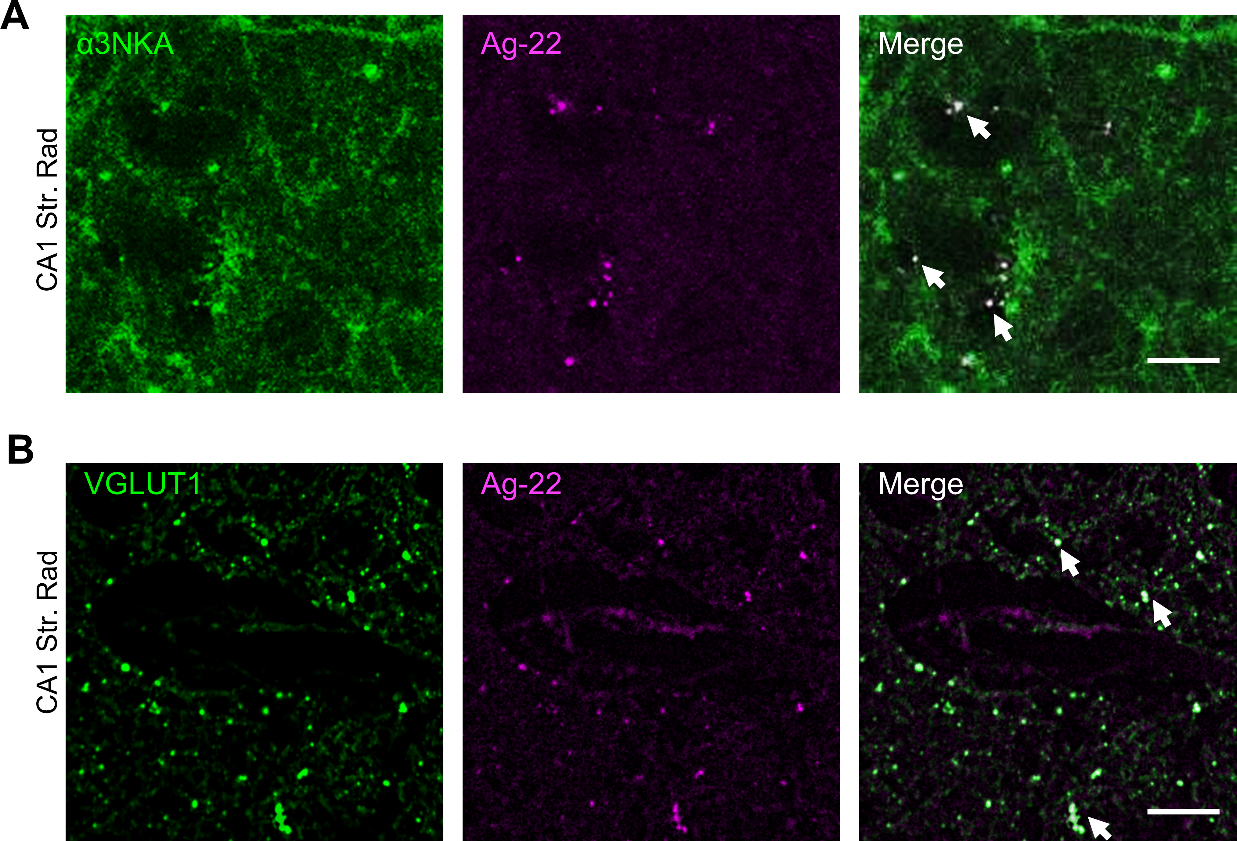
**

**Supplemental Figure S6. Ag22-scarlet colocalizes with the** α**3NKA agrin receptor and with the excitatory presynaptic marker VGLUT1 in juvenile mice.** (A) Agrin-22 binding sites and α3NKA were colocalized, appearing as small puncta distributed over the CA1 *stratum radiatum*. Scale bar, 5 μm. (B) Double labeling with VGLUT1 revealed agrin-22 concentrated at excitatory synapses. Scale bar, 10 μm.

**
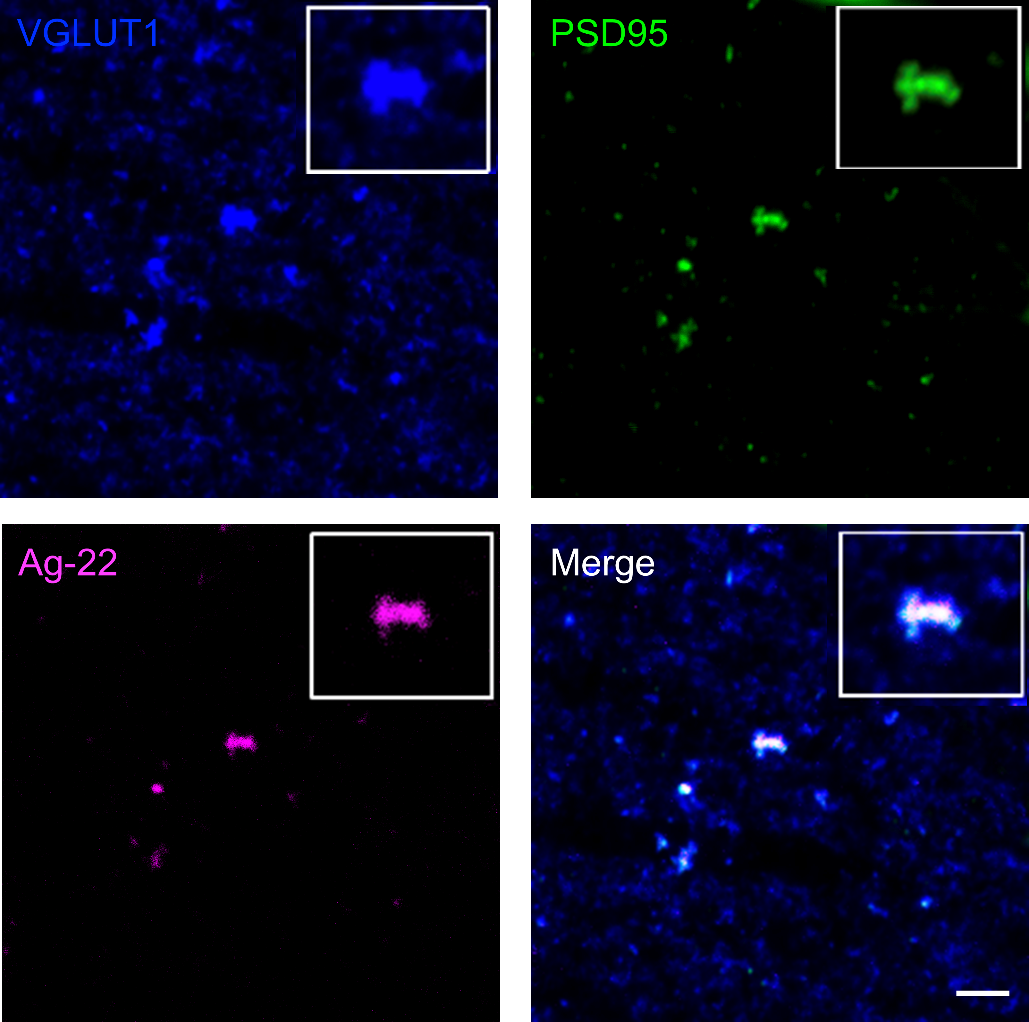
**

**Supplemental Figure S7. Ag22-scarlet colocalizes with the excitatory presynaptic marker VGLUT1 and the excitatory postsynaptic marker PSD95 in complex synapses/synaptic clusters with multiple postsynaptic densities in juvenile mice.** Triple labeling with VGLUT1, agrin-22 and PSD95 revealed agrin-22 concentrated at excitatory synapses. Scale bar, 4 μm.
